# Divide and conquer! Data-mining tools and sequential multivariate analysis to search for diagnostic morphological characters within a plant polyploid complex (*Veronica* subsect. *Pentasepalae*, Plantaginaceae)

**DOI:** 10.1371/journal.pone.0199818

**Published:** 2018-06-29

**Authors:** Noemí López-González, Santiago Andrés-Sánchez, Blanca M. Rojas-Andrés, M. Montserrat Martínez-Ortega

**Affiliations:** 1 Departamento de Botánica y Biobanco de ADN vegetal, Universidad de Salamanca, Salamanca, Spain; 2 Departamento de Didáctica de las Matemáticas y Didáctica de las Ciencias Experimentales, Universidad de Salamanca, Salamanca, Spain; National Cheng Kung University, TAIWAN

## Abstract

This study exhaustively explores leaf features seeking diagnostic characters to aid the classification (assigning cases to groups, i.e. populations to taxa) in a polyploid plant-species complex. A challenging case study was selected: *Veronica* subsection *Pentasepalae*, a taxonomically intricate group. The “divide and conquer” approach was implemented—that is, a difficult primary dataset was split into more manageable subsets. Three techniques were explored: two data-mining tools (artificial neural networks and decision trees) and one unsupervised discriminant analysis. However, only the decision trees and discriminant analysis were finally used to select diagnostic traits. A previously established classification hypothesis based on other data sources was used as a starting point. A guided discriminant analysis (i.e. involving manual character selection) was used to produce a grouping scheme fitting this hypothesis so that it could be taken as a reference. Sequential unsupervised multivariate analysis enabled the recognition of all species and infraspecific taxa; however, a suboptimal classification rate was achieved. Decision trees resulted in better classification rates than unsupervised multivariate analysis, but three complete taxa were misidentified (not present in terminal nodes). The variable selection led to a different grouping scheme in the case of decision trees. The resulting groups displayed low misclassification rates when analyzed using artificial neural networks. The decision trees as well as the discriminant analysis are recommended in the search of diagnostic characters. Due to the high sensitivity that artificial neural networks have to the combination of input/output layers, they are proposed as evaluation tools for morphometric studies. The “divide and conquer” principle is a promising strategy, providing success in the present case study.

## Introduction

Polyploidization is known to have occurred at least once during the evolutionary history of all angiosperms [1,2] and it is widely thought to play an important role in plant evolution and ecology [3–6]. Also, interspecific hybridization may have occurred over plant evolution more frequently than previously suspected [7,8] and in fact involves at least one-quarter of plant-speciation events [9]. Hybridization (including allopolyploidization) and introgression are complex processes that may blur species boundaries in hybrid zones if isolating factors are not definitely established [10,11] and may even end up merging species that were formerly separated [12]. These processes affect species delimitation, giving rise to intermediate phenotypes between the parents [4,13,14], leading to overlapping character states, and many gradual phenotypic transitions (e.g. in related subgenera of *Veronica*, [15]; or in other genera, e.g. Koutecký [16]; Horändl et al. [17] among many others) or results in high intraspecific variation [18–20].

*Veronica* subsect. *Pentasepalae* is a recently diversified complex in which genetic isolation barriers are not definitely established [21–23]. In addition, both polyploidy and hybridization have been identified as processes causing morphological alterations that make species boundaries indistinct and avoid clear-cut recognition of closely related taxa [24]. Consequently, some key aspects remain controversial and/or poorly studied, mainly the determination of species boundaries and the accurate selection of morphological traits to identify them. The complex taxonomy of the study group is reflected in the existence of c. 230 names for just 22 accepted taxa [25]. Although most of the Eurasian species of this group have been reviewed throughout history in partial monographs or taxonomic treatments within several *Floras* (e.g. Watzl [26]; Walters and Webb [27]; Martínez-Ortega et al. [28]), Rojas-Andrés and Martínez-Ortega [25] have proposed the most recent taxonomic treatment for the whole subsection. This taxonomic proposal is based on the results of DNA sequence-based phylogenetic analyses that included all the taxa belonging to the subsection known at that time [22], which are considered together with information on ploidy level, phenotypic characters, habitat preferences, and species distributions. The subsection contains 17 species, four subspecies and one variety [25] and is represented in the temperate regions of Eurasia and in North Africa (only one species). This taxonomic treatment has recently been revised and slightly modified based on AFLP fingerprinting and DNA ploidy-level estimations [23]. This latest taxonomic proposal based on several data sources and on the general lineage concept is taken here as a starting hypothesis (see Materials and Methods). The members of the subsection are characterized by a pentapartite calyx (rarely tetrapartite) with the fifth sepal significantly smaller [22]. Within this subsection some of the taxa are registered on the International Union for the Conservation of Nature Red List (http://www.iucnredlist.org/) and regional catalogues [29], because they are threatened plants with narrow distribution areas and low numbers of known populations [30]. It is necessary to define species boundaries and provide tools to recognize taxa (i.e. useful discriminant characters to be implemented in identification keys), but this is even more important when endangered species are involved.

For identification keys, leaf-lamina shape is one of the most relevant characters; it is remarkably informative for woody plants [31–33]; Kafkas and Perl-Treves [32]; Jensen et al. [33]), but it is also useful to identify species belonging to many other plant groups (e.g. Ackerfield and Wen [34]; Plotze et al. [35]; Andrade et al. [36]). Specifically, the taxonomic treatments available for *V*. subsect. *Pentasepalae* thoroughly consider and use leaves as a primary source of characters for species identification [25–28,37,38], mainly because floral features show little variation in *Veronica* and they are quite ephemeral in comparison to leaf attributes. A previous work that examined leaf variation in eight taxa from the Iberian Peninsula and North Africa demonstrated that an overall separation of taxonomic units was possible based on a set of morphological characters despite some particular cases in which unequivocal identification through these features alone was not accomplished [39].

At present, different methods are available to analyze morphometric data. The classical data analysis through multivariate discriminant analyses (hereafter DAs) are still being successfully applied [40,41]. Multivariate morphometrics represents a robust tool for evaluating variation patterns at the specific and infraspecific levels [42], but new techniques are being implemented and show noteworthy outcomes. Data mining is the core step in the Knowledge Discovery in Databases (KDD), and data-mining tools find and describe structural patterns in data [43]. Data-mining tools have been successfully applied to a broad range of fields such as marketing, chemistry or social studies [44–49]. Although these methods have not been widely used in morphometrics, some examples can be found (see below). Specifically, two well-known data-mining techniques have been previously applied in morphometric studies: “Decision Trees” and “Artificial Neural Networks” (hereafter DTs and ANNs, respectively). DTs are designed to identify patterns defining a given number of different groups, using direct information about the membership of the units [50], which results in classification trees providing decisions at each branch point or node. This technique makes direct use of the “divide and conquer” principle and generates groups automatically while the tree is constructed. DTs have been used in taxonomic and palaeoecological studies involving plant species [51,52]. ANNs, computational models inspired by biological systems, are formed by a number of elements (neurons) organized in layers. Each neuron in a layer is connected with each neuron in the next one by weights, and these weights are adjusted through a learning process (i.e. they are "trained" with respect to specific data until they "learn" the underlying hidden patterns). This technique has lately been used to identify organisms on the basis of morphological traits, mostly in animals [53,54] but not exclusively [55,56]. Also some studies have explored the usefulness of the three previously mentioned approaches in different areas of knowledge and with different objectives, such as species distribution [57], medical data analysis [58], prediction accuracy [59] or disease prediction [60,61]. There is a wide range of data-mining techniques (such as support vector machines, methods based on the K-nearest neighbor algorithm, rule induction, etc.) and statistical methods (e.g. Bayesian approaches, regression-based approaches), but these have been less used for morphometric studies and therefore, are not considered here and thus lie beyond the scope of this work.

The purpose of the present work was to compare the performance of three classification techniques, using the morphologically highly heterogeneous diploid-polyploid complex *V*. subsect. *Pentasepalae* as a case study, applying a “divide and conquer” approach (i.e. a dataset that was difficult to handle was split into more manageable subsets). For this, a search was made for discriminant morphological characters to allow accurate taxon identification in taxonomically intricate species groups. The “divide and conquer” approach has been successfully used for example to align high numbers of DNA sequences [62,63] and phylogenetic analyses using parsimony [64]. The selection of the study group is based on two main criteria that make the case both challenging and robust. First, despite the knowledge acquired after years working on this group, species identification remains problematical; and, second, enough molecular, cytological, biogeographic, and phylogenetic information is available, ensuring a solid starting taxonomic working hypothesis for the reference taxa. Morphometric data have been partially gathered from a previous work by Andrés-Sánchez et al. [39], but this dataset has been substantially augmented (threefold) with information on virtually all the species included in *V*. subsect *Pentasepalae* and, whenever possible, from the entire distribution area of each taxon. For the aim, this work involves the following:

1) Formulation of an optimal classification scheme by assigning cases to groups (i.e. populations to taxa) in accordance with the available taxonomic starting hypothesis. The separation of the entities is forced with the help of subsequent guided DAs. From the leaf features with importance in each DA, the final selection is based on previous knowledge (i.e. manual character selection). This character selection and the initial scheme are used as a reference to be compared with the results found using other techniques (see point 3).
2) Analysis of the morphometric dataset through three techniques at the same level: two data-mining tools [DTs and ANNs, currently available under GNU-GPL license (General Public License)] and an unsupervised systematic multivariate approach. For these methods no previous knowledge is assumed. The analyses are focused on the search for leaf features that are diagnostic for the species (many of them narrowly distributed and with a few known populations) that comprise a recently diversified and morphologically highly heterogeneous plant group affected by hybridization and polyploidization.
3) Assessment of the pros and cons of each approach plus an evaluation of the diagnostic features resulting from each technique. Use of ANNs to determine the suitability of the variables (input layers) over the groupings established (output layers) and comparison with the optimal classification scheme.
4) Verification of whether it is possible to establish an automated protocol to find out diagnostic characters to be readily used in taxon identification keys.

It should be remarked that the purpose of this study is not to achieve automated plant recognition. As stated above, within *V*. subsect. *Pentasepalae*, multiple lines of evidence have previously been used to propose a taxonomic starting hypothesis, following an integrative taxonomic approach [65] and the general lineage species concept of De Queiroz [66,67]; see Rojas-Andrés et al. [22], as well as Padilla-García et al. [23]. Here, well-established taxonomic entities were used as a reference to carry out the main points mentioned here.

## Materials and methods

**Fig 1:**
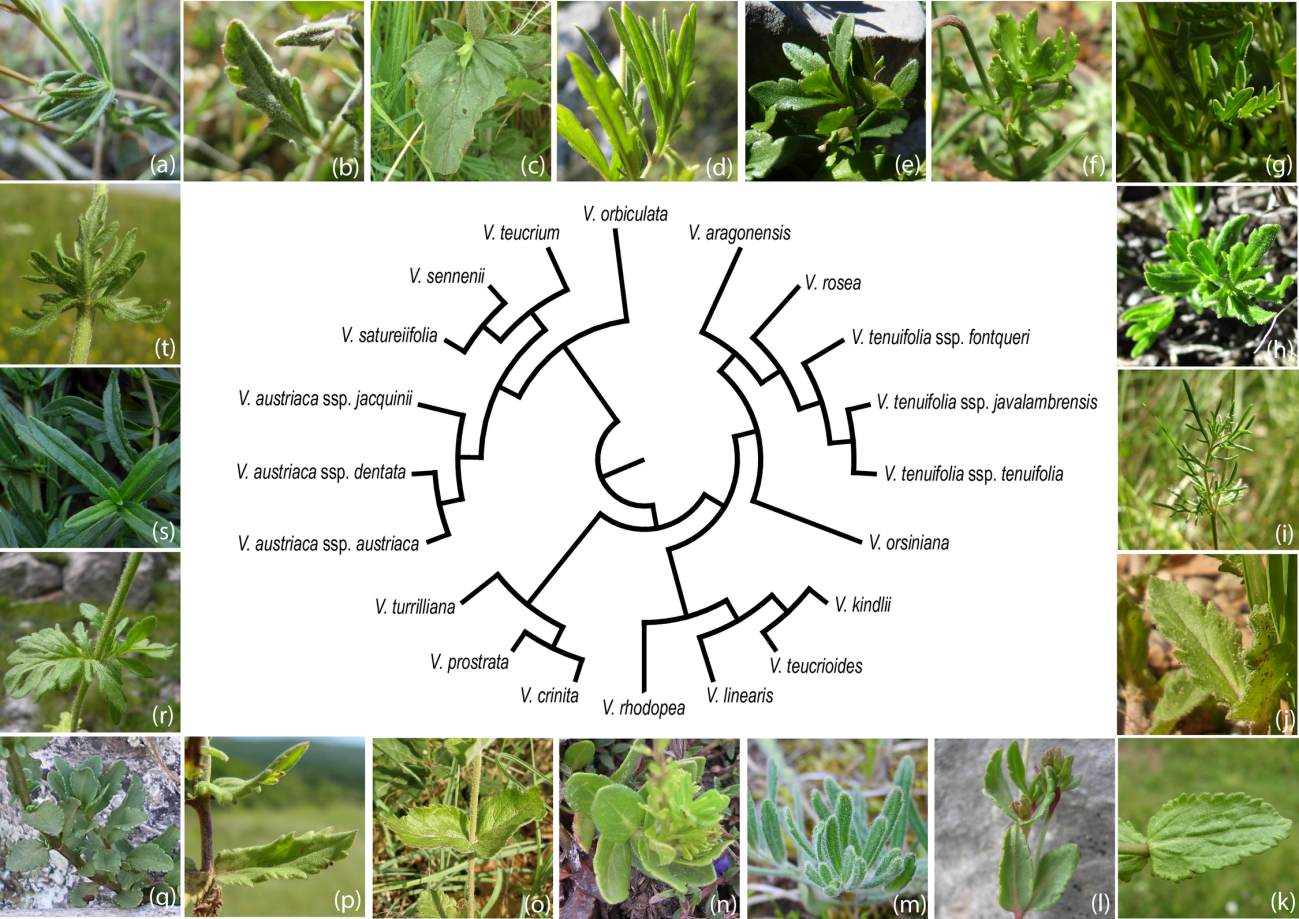
Starting taxonomic hypothesis. Simplified neighbour joining of the taxa examined for *V*. subsect *Pentasepalae*; modified from Padilla et al. 2017. a) *V*. *satureiifolia*, Borau, Spain. Photo: N. Padilla-García; b) *V*. *senneni*, Borau, Spain. Photo: N. Padilla-García; c) *V*. *teucrium*, Novi Sad, Serbia. Photo: S. Andrés-Sánchez. d) *V*. *orbiculata*, Makarska, Croatia. Photo: S. Andrés-Sánchez; e) *V*. *aragonensis*, Mount Baziero, Spain. Photo: N. Padilla-García; f) *V*. *rosea*, Djebel Lakra, Marruecos. Photo: S. Andrés-Sánchez; g) *V*. *tenuifolia* ssp. *fontqueri*, Sierra de las Nieves, Spain. Photo: J. Peñas de Giles; h) *V*. *tenuifolia* ssp. *javalambrensis*, Valdeajos, Spain. Photo: N. Padilla-García; i) *V*. *tenuifolia* ssp. *tenuifolia*, Bordón, Spain. Photo: M. M. Martínez-Ortega; j) *V*. *orsiniana*, Iglesuela del Cid, Spain. Photo: M. M. Martínez-Ortega; k) *V*. *kindlii*, Pljevlja, Montenegro. Photo: S. Andrés-Sánchez; l) *V*. *teucrioides*, Mount Olimpus, Greece. Photo: B. M. Rojas-Andrés; m) *V*. *linearis*, Kozjak Lake, FYROM. Photo: N. López-González; n) *V*. *rhodopea*, Belmeken, Bulgaria. B. M. Rojas-Andrés; o) *V*. *crinita*, Popovitsa, Bulgaria. Photo: M. M. Martínez-Ortega; p) *V*. *prostrata*, Pirot, Serbia. Photo: S. Andrés-Sánchez; q) *V*. *turrilliana*, Veleka river, Bulgaria. Photo: B. M. Rojas-Andrés; r) *V*. *austriaca* ssp. *austriaca*, Cerna Mountains, Romania. Photo: A. Badarau; s) *V*. *austriaca* ssp. *dentata*, Botanical Garden (Univerzity Karlovy, Prague), Czech Republic. Photo: M. Kesl; t) *V*. *austriaca* ssp. *jacquinii*, Josipdol, Croatia. Photo: S. Andrés-Sánchez;

**Fig 2:**
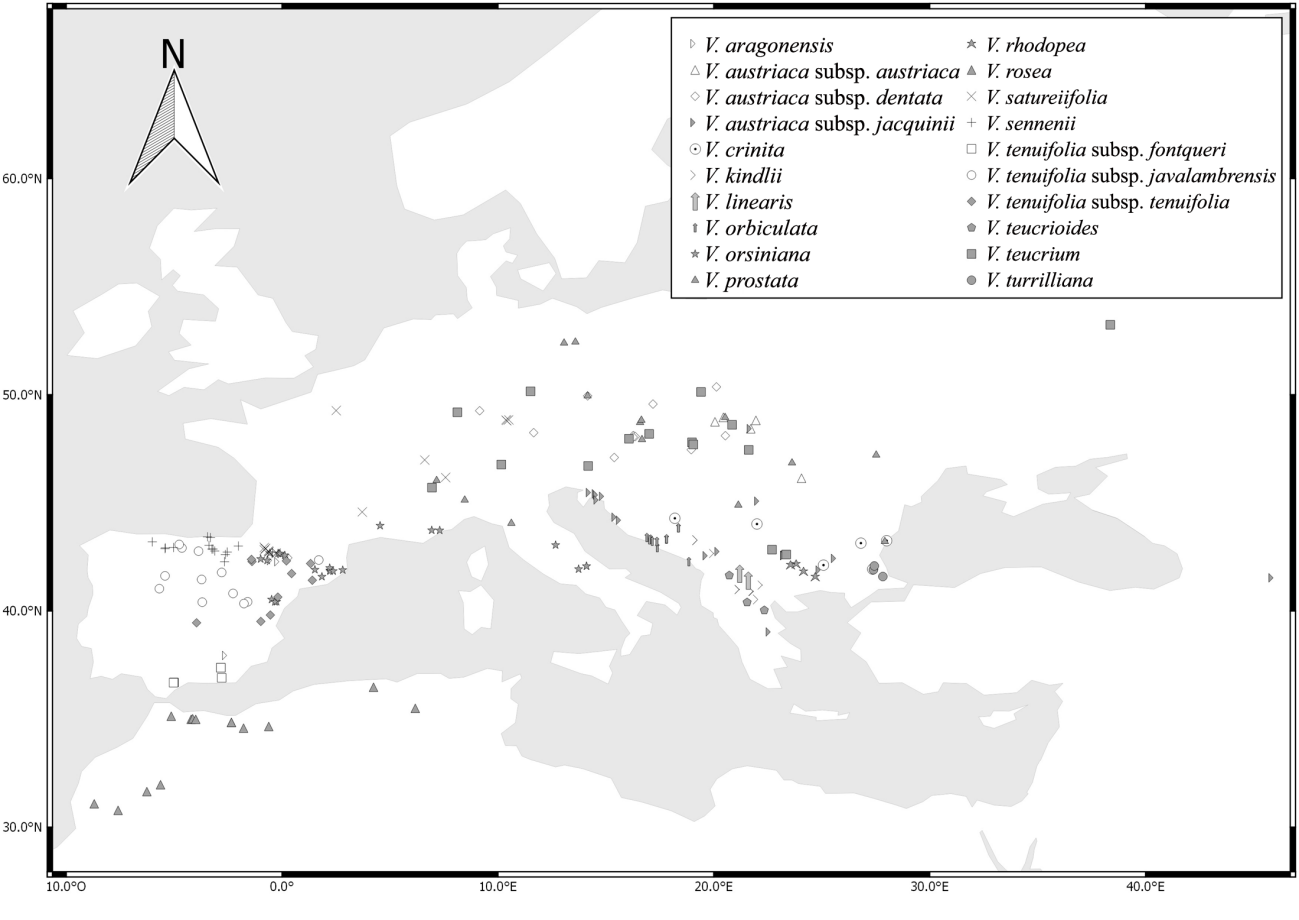
Distribution map of the populations included in this study.

A total of 605 specimens (individuals) from 209 populations were studied, either on loan from 19 herbaria—B, BC, BCF, BM, DR, E, FCO, G, GDA, JACA, K, MA, MAF, MGC, RNG, SEST, SEV, VAB and VIT—or collected during the present study and deposited in SALA (herbarium acronyms according to Thiers, continuously updated [68]). The selection of the material measured was based on the species distribution. The initial attempt was to evaluate all the species and subspecies currently included in *V*. subsect. *Pentasepalae*, but finally three taxa could not be studied for lack of available material (*V*. *krylovii* Schischk., *V*. *thracica* Velen., and *V*. *dalmatica* N.Pad.Gar., Rojas-Andrés, López-González & M.M.Mart.Ort.). Therefore 20 of the 23 species and subspecies comprising the subsection according to Rojas-Andrés and Martínez-Ortega [25], and Padilla-García et al. [23] were examined. Details about the plant material are given in S1 Table ordered according to the initial identification. The number of individuals and populations studied is summarized in Table 1, and the abbreviation assigned to each operational taxonomic unit (OTU) is indicated. The taxonomic starting hypothesis follows Rojas-Andrés et al. [22] and Padilla-García et al. [23], which is based on the results from previous molecular, cytological, biogeographic, phylogenetic and morphological studies (Fig 1 and Table 1). The spatial distribution of the specimens selected is displayed in Fig 2.

**Table 1:** Plant material.

Operational taxonomic unit (OTU)	Number of individuals	Number of populations
*V*. *aragonensis* Stroh. (ARA)	21	7
*V. *austriaca* L.	-	-
(1)*V*. *austriaca* L. ssp. *austriaca* (AUS)	15	5
(2)*V*. *austriaca* ssp. *dentata* (F. W. Schmidt) Watzl (DEN)	36	12
(3)*V*. *austriaca* ssp. *jacquinii* (Baumg.) Watzl (JCQ)	55	19
*V*. *crinita* Kit. (CRI)	25	9
*V*. *kindlii* Adamović (KIN)	24	8
*V*. *linearis* (Bornm.) Rojas-Andrés & M. M. Mart. Ort. (LIN)	6	2
*V*. *orbiculata* A. Kern. (ORB)	33	11
*V*. *orsiniana* Ten. (ORS)	72	24
*V*. *prostrata* L. (PRO)	43	15
*V*. *rhodopea* Degen. ex Stoj. & Stef (RHO)	14	5
*V*. *rosea* Desf. (ROS)	40	14
*V*. *satureiifolia* Poit. & Turp. (SAT)	42	15
*V*. *senneni* (Pau) M. M. Mart. Ort. & E. Rico (SEN)	41	15
*V. *tenuifolia* Asso	-	-
(1)*V*. *tenuifolia* ssp. *fontqueri* (Pau) M. M. Mart. Ort. & E. Rico (FON)	14	5
(2)*V*. *tenuifolia* ssp. *javalambrensis* (Pau) Molero & J. Pujadas (JAV)	34	12
(3)*V*. *tenuifolia* Asso ssp. *tenuifolia* (TEN)	29	10
*V*. *teucrioides* Boiss. & Heldr. (TCR)	9	3
*V*. *teucrium* L. (TEU)	43	15
*V*. *turrilliana* Stoj. & Stef. (TUR)	9	3
Total	605	209

Summary of individuals and populations included in the morphometric study. The abbreviations of the 20 operational taxonomic units (OTUs) corresponding to the taxonomic starting hypothesis are indicated in brackets.

* The species marked with an asterisk comprise several subspecies; those belonging to *V*. *austriaca* have been highlighted in blue, while those of *V*. *tenuifolia* appear in red.

The 30 quantitative characters (abbreviations shown in Table 2) already used in Andrés-Sánchez et al. [39] were measured for the additional taxa and populations included here. Except for cases in which the available material was insufficient, each character was measured in three specimens per population and the arithmetic mean was calculated. The matrices containing raw data and all the average values per population are available on GitHub (https://github.com/NoeLG4/morpho.dataset).

**Table 2:** Characters measured and abbreviations.

Abbreviation	Morphological character		
**LT**	Medium leaf	Length of trichomes	
**DI**		Density of indumentum	
**MLWM**		Width	Maximum width
**WMPM**			Middle part
**TLWM**			Entire terminal part
**FTWM**			First tooth
**STWM**			Second tooth
**LLM**		Length	Total
**FTLM**			First tooth/segment
**LFFM**			First division/segment (bipinnatisect leaf)
**STLM**			Second tooth/segment
**LFSM**			First tooth of the second segment (bipinnatisect leaf)
**PLM**			Petiole
**DBMWM**		Distance between the leaf base and the maximum width line	
**DLAUM**		Distance between the leaf apex and the uppermost teeth	
**NTM**		Number of teeth per hemilimb	
**MLWS**	Leaf of the apical shoot	Width	Maximum width
**WMPS**			Middle part
**TLWS**			Entire terminal part
**FTWS**			First tooth
**STWS**			Second tooth
**LLS**		Length	Total
**FTLS**			First tooth/segment
**LFFS**			First division/segment (bipinnatisect leaf)
**STLS**			Second tooth/segment
**LFSS**			First tooth of the second segment (bipinnatisect leaf)
**PLS**			Petiole
**DBMWS**		Distance between the leaf base and the maximum width line	
**DLAUS**		Distance between the leaf apex and the uppermost teeth	
**NTS**		Number of teeth per hemilimb	

The measurements were taken from a leaf situated in the central segment of the stem (medium leaf) (Fig 2 in Andrés-Sánchez et al. [39]) and from one on the apical shoot (Fig 3 in Andrés-Sánchez et al. [39]). The measurements were taken with a digital electronic caliper Digimatic 500 (Mitutoyo American Corporation, Aurora, USA). Characters related to the indumentum were calculated only in the medium leaves. One measurement was made for each variable except for hair length, for which five trichomes per leaf were considered. “Density” was indirectly estimated by counting the number of hairs on a 1-cm-long linear transect at the leaf margin. Hair length and “density” were determined by means of a stereoscopic zoom microscope NIKON SMZ-U (Nikon Corporation, Tokyo, Japan) equipped with a video camera SONY 3CCD DXC-930P (Sony Corporation, Tokyo Japan). The photos taken were transferred to a computer and examined through the image-analysis software Image-Pro Plus version 1.0 (Media Cybernetics Inc., Rockville, USA).

**Fig 3:**
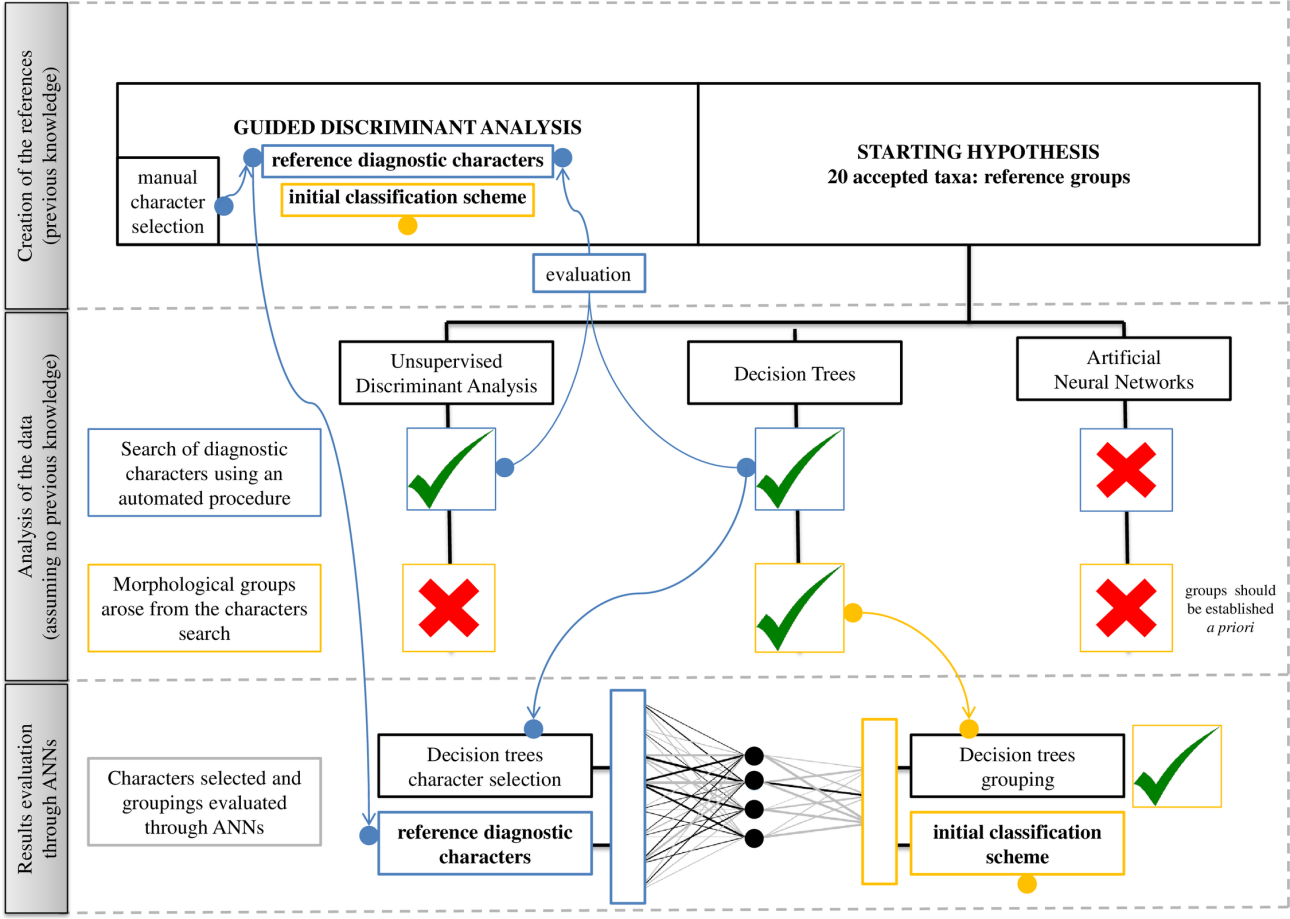
Workflow. The workflow involves the following steps (separated in the image by dashed gray lines): creation of the references, data-analysis approaches and evaluation of the results. The green ticks mean optimal outcomes while red crosses mean suboptimal ones. Processes related to the search of diagnostic characters are indicated in blue, while those corresponding to the groupings are indicated in light orange.

In an effort to avoid the size effect, some characters were considered as quotients (LLM/MLWM, LLM/WMPM, LLM/DBMWM, FTLM/FTWM, STLM/STWM, DLAUM/TLWM, LLM/DLAUM, LLS/MLWS, LLS/WMPS, LLS/DBMWS, FTLS/FTWS, STLS/STWS, DLAUS/TLWS and LLS/DLAUS).

The absence of normality was checked and the Spearman correlation coefficients were determined from the original matrix of descriptors in order to test for correlation between primary variables. The primary matrix was reduced by removing one of the variables shown to be correlated for all subsequent analyses; the threshold applied was 0.95. Statistical analyses were performed using the open-source R platform (descriptive statistics, Spearman correlation) [69].

A Euclidean coefficient was used to compute the secondary distance matrix after standardization of the characters in the primary matrix. Then, a principal component analysis (PCA) was performed with no *a priori* knowledge of the population groupings (i.e. ordination of the OTUs as revealed by leaf characters). Computations were made with the software NTSYSpc 2.21n [70].

Fig 3 illustrates the data-analysis approach followed, which is described below.

### Building an initial classification scheme based on the taxonomic starting hypothesis: Guided discriminant analyses

Several canonical discriminant analyses (DAs) were performed using the software SPSS v. 15 for Windows (SPSS, Chicago, USA) over the standardized variables, which were selected manually to force the separation of each of the previously accepted taxonomic units and to provide an initial reference classification scheme.

Four sequential DAs were conducted for the division of the initial data set into smaller subsets and therefore simplify its complexity. This was done by selecting one of the most discriminant characters derived from the original and subsequent DAs (i.e. those based on the initial data set and different subsets established in further steps; see Results section). Character selection was manual, based on previous knowledge of the species group. The variables finally employed were: STLM/STWM (which divides the taxa into specimens with medium leaves entire to pinnatifid vs. pinnatipartite to bipinnatisect; *sensu* Beentje [71]), DI (densely hairy leaves vs. subglabrous to glabrous leaves), LT (short vs. long trichomes) and LLM (to distinguish taxa showing large medium leaves from those with small medium leaves). By this character selection, some phenotypic groups arise. Within these final groups, several characters were further used, forcing taxon classification. To show the variability of the selected characters within each species in a comparable way, graphic tests (i.e. box-plot with indication of median values) were conducted. The box-plots were generated using the “ggplot2” package in R [72]. Following this procedure, some particular observations (populations) were classified as belonging to a taxon that did not match the initial identification. These observations were considered errors. The misclassification rate (MCR: number of misclassified cases regarding the total) was calculated as the sum of errors (i.e. the misclassified cases) in each division. A misclassification in a superior division forces an observation to be misguided and never reach correct classification.

### Searching for diagnostic characters assuming no previous knowledge

The purpose of the data analysis was to search for leaf features that would be diagnostic for the species. This search was assumed to be uninfluenced by prior knowledge of the group, meaning that manual intervention or decisions based on previous knowledge should be ruled out. For the implementation of the “divide and conquer” approach, the character selection should reduce the complexity of the initial dataset, recurrently dividing this initial matrix into subsequent subgroups (i.e. generating a grouping scheme).

#### (1) Unsupervised discriminant analyses (unsupervised multivariate analysis)

Unsupervised multivariate analysis discarding manual intervention was carried out. For this, several canonical discriminant analyses (DAs) at different scales were performed using the software SPSS v. 15 for Windows (SPSS, Chicago, USA) following a systematic, sequential approach. The procedure was unsupervised, assuming some artificial criteria to rule out manual intervention and thus decisions based on previous knowledge. This was done by selecting the variable showing the highest percentage of variance explained in the first discriminant function in each DA. This character was then represented in a box-plot, allowing the separation of the dataset into two subsets. Once the variable was chosen, the threshold for splitting the data was established according to two conditions: (1) the main bodies of the box-plots could not overlap, and (2) the threshold should minimize the number of misclassified cases for each step. This procedure was recurrently applied until every species and subspecies was individually classified. The misclassification rate was calculated as explained in the previous section. The box-plots were generated using the “ggplot2” package in R [72].

#### (2) Decision Trees

DTs have a built-in mechanism for performing variable selection [73]. This technique explicitly focuses on relevant features while ignoring irrelevant ones [74], so that there is no need of prior feature selection. Together with feature selection, the treatment of missing data is a key issue to be considered during the pre-processing of the data when working with data-mining tools. Due to the low number of missing cases in the present study, the only population presenting them was removed from both DTs and ANNs. First of all, a perfect tree that fits the data was produced, setting the minimum size of the terminal node to the minimum number of observations in the dataset (two, as it is indicated below) and the minimum residual deviance to zero. These parameters enable the tree to detect taxa even with only two cases (populations) available (e.g. *V*. *linearis*, see Table 1) and classify all the observations (if the limit on tree depth allowed it), but this tree is clearly over-fitted (see Results) and therefore useless. The tree was grown by binary recursive partitioning.

The splitting criterion is the division that maximizes the reduction in deviance; splitting continues until the terminal nodes are too small or too few to be split [75]. These kinds of trees lead to a large number of terminal nodes and are usually over-fitted, so in a second step the tree was simplified by “pruning” [76]. This technique reduces the initial size by removing the least important splits. The classification trees and the parameters to evaluate them (residual mean deviance and misclassification rate) were taken directly from the package “tree” in R [75]. The procedure for calculating the misclassification rate is analogous to that of DAs: it results from the sum of the misclassified cases in each node. However, in this case a misclassification in a higher division does not necessarily force an observation to be misguided because some taxa appear in more than one final node.

The script used to analyze the data is available on GitHub (https://github.com/NoeLG4/morpho.DT).

#### (3) Artificial neural networks

Feature selection when working with ANNs is a critical step [43]. Perfectly correlated variables are truly redundant, meaning that no further information is gained by adding them [77]. Therefore, correlated variables were removed from the dataset and all remaining features were initially considered. Most of the variables considered in the present study were leaf measurements so that some degree of correlation was expected. Furthermore, some of them were highly correlated with each other (>0.8), making the task of selecting sufficient independent variables especially difficult. With this taken into account, the determination of the best conditions for the ANN was performed by a preliminary test among several ANNs with different configurations of variables in combination with inspections of time-series plots of potential inputs and outputs [78]. Max-Min standardization was carried out to ensure that each input variable received the same attention [78]. The output layers (representing the taxa) were transformed into binary variables through effect coding. The algorithm used by the ANN for its training was designated by “rprop+” (resilient backpropagation with weight backtracking [79]). All neural networks were performed using the “neuralnet” package [80] included in R.

Once the input layers were established, several networks were performed with 50%, 60%, and 70% of the cases randomly chosen as training data (and the rest reserved for testing the models), with different number of hidden layers (1, 2), and different number of neurons within each hidden layer (from 8 to 16). Because ANNs are sensitive to subtle changes [81] three different training datasets were generated for each analysis. With the use of these three datasets, the parameters were established (percentage of training set, number of hidden layers and number of neurons). With the parameters fixed, 10 different training and test sets were created and the total and per species misclassification rates were then calculated as the average of incorrectly assigned examples in the distinct test sets. Analyses including the 20 taxa resulted in high misclassification rates (see Results) so that 10 random groups were generated for four categories of output layers: 4, 8, 12, and 16 (i.e. 4, 8, 12, and 16 species or infraspecific taxa) to evaluate the performance by number of species. The parameters ‘percentage of training set’ and ‘number of hidden layers’ remained constant, the number of neurons changed in each case to optimize the outcomes. A neural interpretation diagram (hidden layers = 1; neurons = 8; output layers = 8) is shown in S1 Fig. This general scheme of a typical three-layered ANN architecture was produced using the R function plot.nnet [82]. The graphic displaying a misclassification rate by the number of species (S2 Fig) was calculated through “ggplot2” package [72]. The script used for analyzing the data and generating the graphics is available on GitHub (https://github.com/NoeLG4/morpho.ANN).

### Artificial neural networks as a tool to evaluate morphological groups established through a set of specific features

Since only suboptimal results were found when the whole taxonomic group was considered, ANNs were finally not used for the initial aim. However, taking advantage of the high sensitivity of this technique to the combination of input and output layers, they were used to assess the capacity of the selected variables to classify the taxa within the final groups established by the best technique (see Results). This procedure was also used with the variables selected with the help of the guided DAs and the corresponding groupings (initial identification scheme) to be used as reference. The variables used in guided DAs and DTs were selected as input layers for ANNs, and the different final groups established with these techniques (see Results) were treated as output layers. The number of neurons was set and the misclassification rate calculated as explained in the previous section. The analyses were made using the “neuralnet” package [80] included in R.

## Results

The results of the PCA (Table 3) indicate that the variance of the data is explained mostly by the selected morphological variables. The first, second, and third components accounted for 53.57%, 17.04%, and 7.86%, respectively, of the total variation among populations. Nevertheless, due to the high number of observations a clear structure is not evident in the corresponding graphic (figure not shown).

**Table 3:** Principal component analysis.

Axis	Eigenvalue	Percent	Cumulative
1	696.91	53.57	53.57
2	221.66	17.04	70.61
3	102.31	7.86	78.47
4	69.06	5.31	83.78
5	52.20	4.01	87.79
6	31.60	2.43	90.22
7	22.05	1.70	91.92
8	21.46	1.65	93.57
9	16.39	1.26	94.83
10	15.23	1.17	96.00

Eigenvalues and percentages of the data variance accounted by each axis.

The Spearman correlation coefficients calculated from the original matrix of descriptors showed that some of the primary characters were highly correlated (> 0.95). The pairs MLWM-WMPM, LLM/WMPM-LLM/MLWM, LLS/MLWS-LLS/WMPS, and MLWS-WMPS displayed the following values: 0.971, 0.961, 0.955, and 0.953, respectively. Therefore, the variables MLWM, LLM/WMPM, LLS/MLWS, and MLWS were excluded from all subsequent analyses.

### Initial classification scheme through guided discriminant analyses

**Fig 4:**
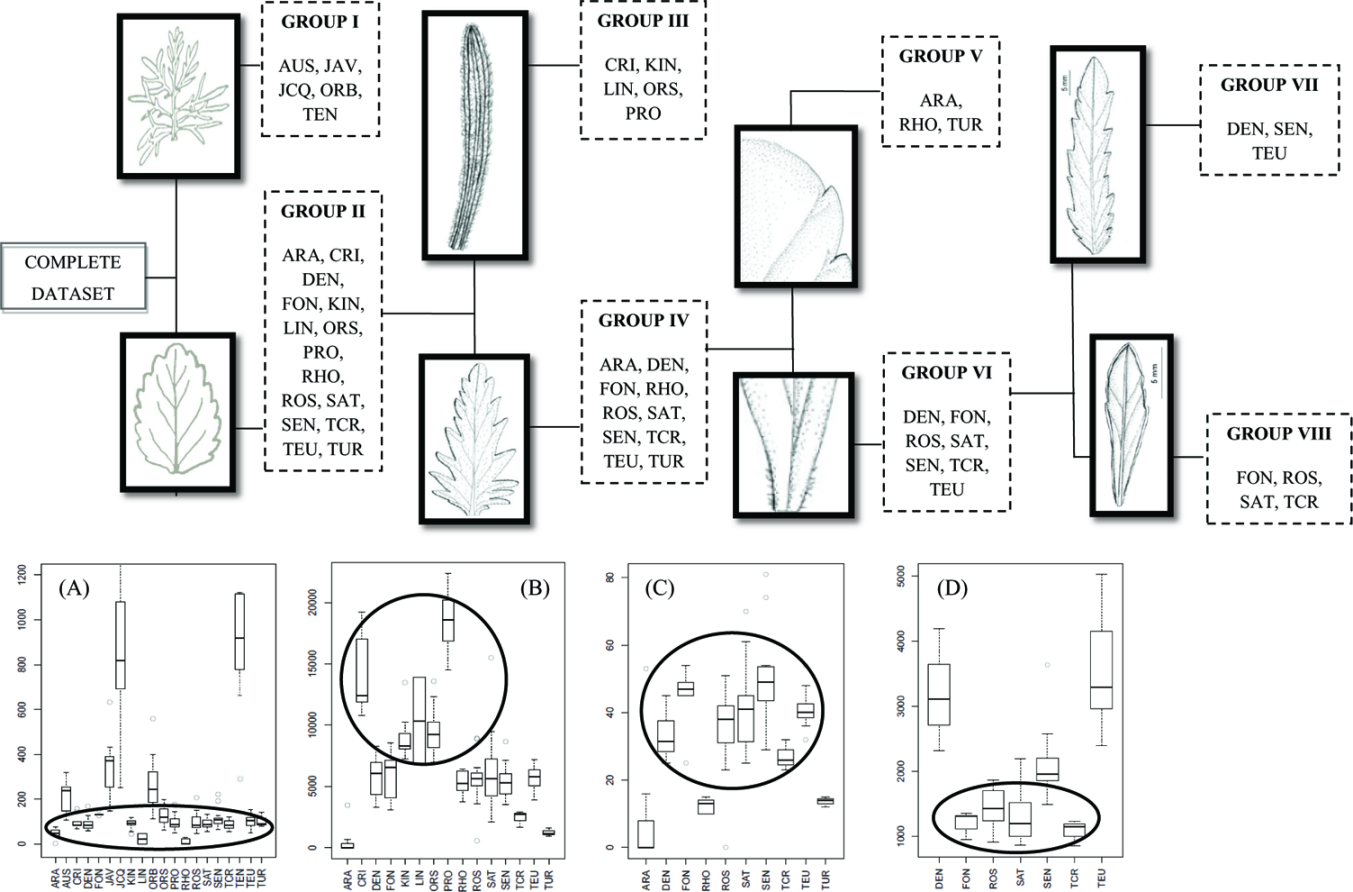
Initial classification scheme through guided DAs. Partition of the original dataset in accordance with the starting hypothesis. Box-plots for (A) STLM/STWM, (B) DI, (C) LT, and (D) LLM. See Table 1 for abbreviations. The circles indicate Group II, Group IV, Group VI, and Group VIII, respectively.

An initial DA performed using the original data matrix showed that a set of variables contributed highly to the discriminant functions and therefore could be selected to delimit the first two sub datasets. Some of these features (i.e. STLM, FTLM, STLM/STWM, FTLM/FTWM, STLS, and FTLS) were related to leaf division (Fig 4A). There was another set of variables not related to leaf division (i.e. NTM, WMPM, and LLM) that could also be used to delimit the first two sub-datasets. Among these two sets of variables, those related with leaf division were considered more informative, and consequently STLM/STWM was finally selected. Following this procedure, subsequent DAs applied to different subsets of species showed sets of features that could be selected for the recursive partitioning of the dataset. Among these variables DI, LT, and LLM were chosen. The selection of these variables reduced the complexity of the dataset even if these features did not contribute the most to the discriminant functions (all discriminant functions, standard coefficients, and structure matrix tables are shown in S2 Table). The subsequent partitions of the original dataset into subsets of species (Groups I to VIII) are displayed in Fig 4, together with box-plots for the chosen leaf characters corresponding to sequential DAs that maximize differences between subsets. These eleven variables used for species and infraspecific taxon classification constitute the reference diagnostic characters (STLM/STWM, DI, LT, LLM, STWM, LLS/WMPS, NTS, STLM, PLS, FTLM and WMPM). This initial classification scheme has a misclassification rate of 0.18 (38/209).

Group I holds the taxa with pinnatipartite to bipinnatisect medium leaves, while Group II contains the species with entire to pinnatifid medium leaves. Within Group I the quotient STLM/STWM (Fig 4A) helps to differentiate between [JCQ+TEN] and [AUS+JAV+ORB]. STWM further helps to distinguish JCQ from TEN, while LLM and LLS/WMPS differentiate among AUS, JAV, and ORB [all box-plots corresponding to Group I are shown in S1 File, from (a) to (d)].

The next partition within Group II was made using the character DI, which renders Group III (taxa showing pubescent leaves), and Group IV (taxa with subglabrous leaves) (Fig 4B). Within Group III, NTS is most helpful to distinguish among CRI and [LIN+KIN+ORS+PRO] and STLM values do not overlap between LIN and the rest. Furthermore, PRO is easily distinguishable from [KIN+ORS] based on the character DI, and KIN can be differentiated from ORS based on the character LT [all box-plots corresponding to Group III are shown in S1 File, from (e) to (h)].

Within Group IV, the next partition is based on the length of the trichomes (LT; Fig 4C). Thus, two further groups resulted: Group V, which contains the species bearing short trichomes on their leaves, while Group VI includes taxa having long ones. Within Group V, ARA can be distinguished from [RHO+TUR] using the character PLS. Moreover, FTLM could be used to separate RHO from TUR [box-plots corresponding to Group V are shown in S1 File, (i) and (j)].

Finally, the taxa included in Group VI could be separated into two further subgroups based on the character LLM (Fig 4D): While Group VII included the entities having medium-sized and long leaves, Group VIII comprised the taxa with small leaves. Within Group VII, SEN could be differentiated from [DEN+TEU] (and even from the remaining taxa within Group VI) based on this character [LLM; S1 File, (k)]. Additionally, TEU differed from DEN in the width of their medium leaves (WMPM) [box-plots corresponding to Group VII are shown in S1 File, (k) and (l)]. Within Group VIII, the characters STWM help distinguish ROS from [FON+SAT+TCR]; moreover, the variable STLM registered values that did not overlap between FON and [SAT+TCR], and these two species could be easily differentiated using the variables DI or DLAUM [all box-plots corresponding to Group VIII are shown in S1 File, from (m) to (o)].

### Searching for diagnostic characters assuming no previous knowledge

#### (1) Unsupervised discriminant analyses

Ten variables were used for species and infraspecific taxon classification through unsupervised DAs (STLM, WMPM, NTM, FTLM/FTWM, DI, LT, LLM, STWM, LLM/DBMWM and STLS/STWS). All the divisions performed with the most discriminant characters determined by DAs are available in S2 File. The first DA carried out indicated that the variable STLM gave the highest percentage of variance explained in the first discriminant function according to the structure matrix (all discriminant functions, standard coefficient, and structure matrix data are shown in S3 Table). In this first step [JCQ+TEN] were separated from the rest of the taxa; additionally, the variable identified to distinguish JCQ from TEN was WMPM. The best character according to the next pair of DAs was NTM, which separated in one step [ARA+LIN+RHO] from the rest of taxa and in the second one, the remaining species from CRI. Within the above-mentioned group, FTLM/FTWM helped to distinguish [ARA+LIN] from RHO, and DI separates ARA from LIN. The fourth DA applied revealed WMPM as the best character and led to the distinction of TEU from the other taxa. The leaf feature showing the highest percentage of variance explained in the first discriminant function suggested by the next discriminant analysis was DI, which applied to the subset separates [AUS+KIN+ORS+PRO] from the remaining taxa (nine at this point). DI arose again as the best variable to differentiate PRO from [AUS+KIN+ORS]; LT allowed the separation of ORS from [AUS+KIN], finally AUS could be distinguished from KIN based on LLM.

LLM was used again in the subsequent DA to distinguish [DEN+SEN] from the remaining taxa. This character was also useful to differentiate DEN from SEN. In the next DA the best variable found was STWM, and the threshold with the fewest misclassified cases split the current subset into [TCR+TUR] and the rest of the taxa; additionally, LT was found to be the best feature to differentiate between TCR and TUR. At this point, only five taxa remained; the DAs performed to distinguish among them gave as a result STWM, LLM/DBMWM, and STLS/STWS as the most discriminant characters, separating ROS, JAV, and SAT from [FON+ORB], in this order. The best variable emerged for the last DA was DI, with slightly higher values in FON.

Through this variable selection in most of the steps, just one or two species were separated from the rest. This process generated a greater number of groups than did guided DAs, with a low number of species in each group. The complete sequence of DAs had a misclassification rate of 0.33 (68/209).

#### (2) Decision trees

The perfect tree correctly classified all the observations (residual mean deviance = 0; misclassification error rate = 0), but led to 48 final nodes (S3 Fig). Some of the species (e.g. ROS, SAT, SEN) appeared more than three times, highly over-fitted, considering that the dataset contained a total of 20 taxa.

**Fig 5:**
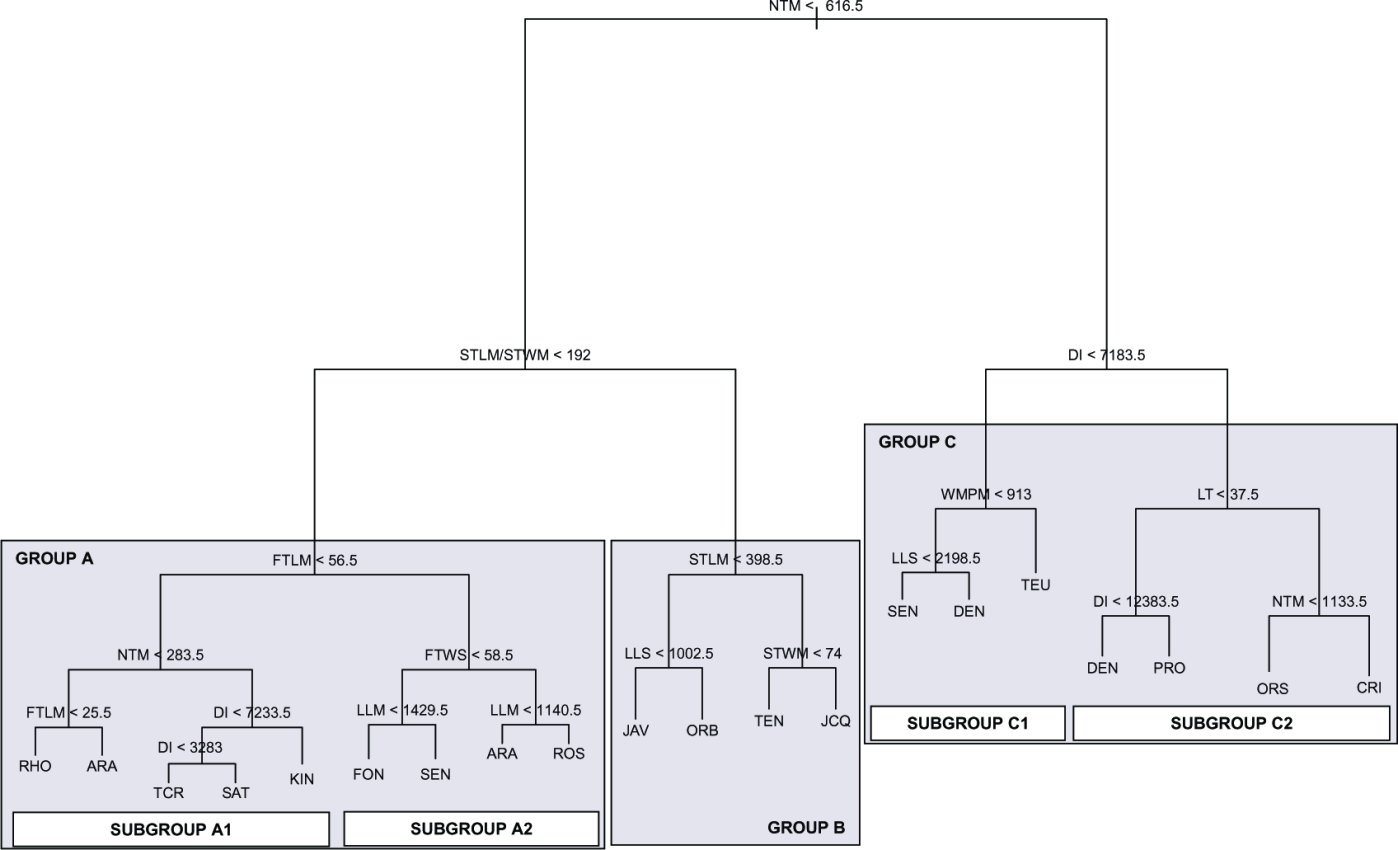
Pruned tree.

The pruned tree (Fig 5) showed 20 terminal nodes, which did not correspond exactly to the 20 taxa: 14 entities were identified corresponding to a single terminal node, three were found in two terminal nodes of separate branches (ARA, DEN, SEN), and three complete taxa were misidentified (AUS, LIN, TUR). The residual mean deviance was 0.998 and the misclassification rate 0.19 (40/208; one observation was eliminated due to lack of information; see Materials and Methods). In tree construction, 11 variables were actually used (STLM/STWM, STWM, STLM, DI, LT, NTM, WMPM, LLS, FTLM, LLM, and FTWS). According to this feature selection, the taxonomic entities can be classified into three main groups (characters and exact values that lead to this classification are shown in Fig 5):

1) Group A [ARA+FON+KIN+RHO+ROS+SAT+SEN+TCR] has low values of NTM and the ratio STLM/STWM. This first group can be subdivided by the character FTLM into Subgroup A1 [ARA+KIN+RHO+SAT+TCR], with shorter teeth, and Subgroup A2 [ARA+FON+ROS+SEN], with longer teeth. 2) Group B [JAV+JCQ+ORB+TEN], has low values of NTM and high for the quotient STLM/STWM, which means pinnatipartite to bipinnatisect leaf. 3) Group C [CRI+DEN+ORS+PRO+SEN+TEU], has high values of NTM; this group can also be subdivided into two subgroups based on DI: Subgroup C1 [DEN+SEN+TEU], with low values; and Subgroup C2 [CRI+DEN+ORS+PRO], with high values of DI.

### Artificial neural networks

From the initial set of variables, 20 were finally selected for ANNs (LT, DI, PLM, STLM/STWM, LLM, WMPM, DBMWM, DLAUM/TLWM, NTM, PLS, STWS, DLAUS/TLWS, LLS/DLBMWS, LLM/DBMWM, TLWS, NTS, LLS/WMPS, LFFM, LFFS, and LFSS). The different percentages used for splitting the data into training and testing sets showed no significant differences in terms of misclassification rate. In each scenario, one hidden layer displayed better outcomes than two or more and heavily reduced the computing time. The number of neurons which resulted in higher predictive capacities varied between 8 and 16, depending on the number of output layers.

The analyses including all taxa led to high misclassification rates (mean value of 0.41, see S2 Fig). Even using the best model, nine of the 20 taxa displayed values over 0.40 (Table 4). Reducing the number of output layers resulted in better values. As shown in S2 Fig, ANNs perform well with groups of less than eight taxa (values of correctly classified cases above 0.75, i.e. a misclassification rate below 0.25), even though it depended largely on the combination of species and infraspecific taxa.

**Table 4:** ANNs per species results.

Species	MCR	NC
**ARA**	0.39	7
**AUS**	1.00*	5
**DEN**	0.38	12
**JCQ**	0.17	19
**CRI**	0.89*	9
**KIN**	0.56*	8
**LIN**	0.33	2
**ORB**	0.49*	11
**ORS**	0.18	24
**PRO**	0.10	15
**RHO**	0.33	5
**ROS**	0.56*	14
**SAT**	0.30	15
**SEN**	0.55*	15
**FON**	0.44*	5
**JAV**	0.05	12
**TEN**	0.17	10
**TCR**	1.00*	3
**TEU**	0.10	15
**TUR**	1.00*	3

(Hidden layer = 1, number of neurons = 16, output layers = 20). MCR = misclassification rate. NC = number of cases.

* Values over 0.4 indicated with an asterisk.

### Comparison of the variables and groups resulting from guided DAs and DTs. Evaluation of the classification power with ANNs

Of the three techniques applied in the present study: ANNs gave suboptimal results when applied to the total dataset, unsupervised DA failed to generate manageable groups, and DTs offered good results regarding both aspects. Therefore, the leaf characters selected through DTs (and the corresponding groupings) were compared to those manually selected with the help of guided DAs (and the initial classification scheme) in terms of misclassified cases using ANNs. Some groups displayed high misclassification rates (Group VIII for DAs: 0.36; Group A and Subgroup A1 for DTs: 0.33 and 0.39, respectively), but the overall misclassification rate was below 0.2 in most cases, as shown in Table 5 (guided DAs variables and groups) and Table 6 (DTs variables and groups).

**Table 5:** MCR calculated through ANN.

	Guided DAs final groups; hidden layer = 1				
**Neurons**	**8**	**10**	**10**	**8**	**12**
**Group**	I	III	V	VII	VIII
**Error**	0.14	0.053	0.024	0.038	0.089
**Reached threshold**	0.0086	0.0089	0.0085	0.0086	0.0086
**Steps**	81.00	64.80	27.36	47.34	86.38
**MCR**	**0.14**	**0.12**	**0.17**	**0.09**	**0.36**

In this case the input layers are the variables manually selected with the help of guided DAs and the output layers the entities within the final groups (initial classification scheme).

**Table 6:** MCR calculated through ANN.

	DTs final groups; hidden layer = 1						
**Neurons**	**18**	**10**	**8**	**12**	**8**	**10**	**12**
**Group**	A	A1	A2	B	C	C1	C2
**Error**	0.153	0.177	0.05	0.109	0.057	0.037	0.041
**Reached threshold**	0.0089	0.009	0.0088	0.0089	0.0087	0.0089	0.0089
**Steps**	321.04	161.49	106.33	101.53	107.64	53.02	50.62
**MCR**	**0.33**	**0.39**	**0.21**	**0.16**	**0.10**	**0.09**	**0.03**

The input layers are the variables selected in DT analysis and the output layers the entities within the final groups obtained through the pruned tree.

## Discussion

This work presents an extensive morphometric analysis focused on relevant leaf characters that covers the complete geographical range of *V*. subsection *Pentasepalae*. A classical multivariate technique (DA) and two data-mining techniques (DTs and ANNs) are used to facilitate the search for discriminant characters and formulation of morphological groups within taxonomically complex plants in which polyploidy and hybridization are involved. A phenotypic species concept is of crucial importance for taxon identification, especially in the field, where other kinds of evidence such as genetic data is still difficult to use. However, when allopolyploidy is involved, morphological data could easily disagree with other sources of information, such as genetic, biogeographic or cytological data, thus complicating the implementation of integrative taxonomy. In this common situation, the methodology used when working with angiosperm species could help to transfer a taxonomic hypothesis based on several lines of evidence to the description of “morphological groups”.

### Generating morphological groups through feature selection

#### (1) Morphological groups based on manual selection

As an initial step in this work, a classic multivariate analysis combined with guided recursive partitioning (guided DAs) was used to establish “morphological groups” as an optimal classification scheme, based directly on prior knowledge of the subsection and on an initial taxonomic working hypothesis [25] (Fig 1). DA is a powerful technique for examining differences among groups with respect to determining whether meaningful differences exist between them [42]. DA finds discriminant functions that best differentiate predefined groups by maximizing the differences between groups while minimizing variation within groups [83]. In the present case, a “divide and conquer” strategy was used through DAs in a directional way. For the implementation of this strategy and for the establishment of “morphological groups” (i.e. I to VIII, Fig 4), the original dataset was sequentially split into subgroups using the most informative variable among those with high absolute correlation within any discriminant function. The misclassification rate was low, but not zero, due to the particularities of the data set, mainly because it included taxa having high levels of phenotypic variability (e.g. allopolyploid taxa).

#### (2) Using DTs to establish morphological groups automatically

For establishing morphological groups in an automated way, DTs are appropriate. DTs (and ANNs) learning methods are part of the KDD process (knowledge discovery on databases), whose final aim is the search for patterns in huge databases [43]. This technique implements the “divide and conquer” principle itself, and the generation of groups based on features is completely automatic. Furthermore, the built-in mechanism of the trees automatically selects the most important variables [73]. DTs therefore are highly useful for effective and, at least, fast initial approximations, because no previous knowledge on the group is required and successful results are achieved even with closely related species [84–85]. The resulting trees display the root at the top. Each sequential division shows an annotation in the graphic output representing the splitting criterion. Cases meeting the criterion go left and those failing to do so go right. The size of the branch above each division shows the decrease in deviance associated with that split. Therefore, the first divisions have longer branches than do the last ones and the branch length diminishes with the depth of the division.

This approach has many advantages: feature selection is intrinsic to the methodology, data transformation is unnecessary, classification success does not depend on the data meeting normality conditions or covariance homogeneity, and the non-linear effect of explanatory variables can be handled [86]. Moreover, some studies reveal that DT analyses perform better on data-sets with incomplete records [87].

Decision trees also have drawbacks. They can be unstable and small changes in the training data can result in alterations in the final tree [88], but this is not a serious disadvantage, being easily solved by bootstrapping [89,90]. Another problem is the method’s inability to manage groups with low numbers of cases unless a perfect tree is produced. This generates an over-fitted tree which leads to a useless classification. When a large reference sample is available, DTs are an appropriate choice [51,91]. Consequently, extending the observations in the initial dataset could provide more accurate results with this technique. The main limitation arises when information on these species cannot be added due, as in this particular case, to the small number of existing localities and individuals for some species.

#### (3) Using ANNs for evaluating the "morphological groups" through the combination of variables selected

The ANNs used in this study are based on adaptive learning algorithms (backpropagation algorithms) and are the most widely used type. They consist of an input layer (with neurons representing input variables), an output one (with neurons representing the dependent variables), and one or more hidden layers containing neurons intended to capture the nonlinearity in the data [92]. These networks are versatile and can be used for data modelling, classification, forecasting, control, data and image compression, and pattern recognition [93]. They can handle a great array of data types and integrate them into categorized outputs which can represent nearly anything, from medical diagnoses [94] to echolocation calls in bats [95].

ANNs also have limitations. They can handle various types of data, but for modelling data of low dimensionality, ANNs perform worse than do conventional statistics. On the other hand, they may be used when higher accuracy is required [92]. The data pre-processing is not straightforward and represents a critical step [43], and consequently it has a significant effect on the final model performance [78]. Another drawback is that using ANNs does not allow to direct selection of the most important variables and does not provide p-values for testing the significance of the parameter estimate [96]. However, in this case it should be taken into account that there are some approaches which allow the assessment of the contribution of variables to the model [97]. Another disadvantage usually attributed to the traditional ANNs is a limitation on the generalization of the results that can over-fit the data [98].

In any case, their power as classification tools is beyond doubt. The reason why ANNs are not an appropriate approach in this case is related to the characteristics of the dataset generated by the study group (i.e. too many entities to classify, too few observations per taxon in some cases and the fact that some are highly polymorphic). Probably, ANNs combined with another kind of initial dataset would provide better outcomes. For example, computer-based image analysis has excellent potential even for identification at varietal levels in some plant groups (wheat: Dubey et al. [99]; *Camellia sinensis*: Pandolfi et al. [55]) or when sufficient information about each output case is available (onion varieties: Rodriguez Galdon et al. [100]).

It bears highlighting that ANNs were not used in this work as a classification method, but rather to assess each of the final morphological groups with respect to the variables leading to these groups. ANNs were employed to compare the groups established using DTs and groups formed by a guided DA using the set of characters previously defined by each technique. The properties of this construction (ability to capture hidden patterns in data, good results when accuracy is needed) were taken advantage of together with the good results displayed when dealing with small groups of species (the sensibility to the set of input/output layers observed during this work). ANNs easily adjust to any set of input-output patterns and through a robust training process perform a model function with the minimum possible error. For all these reasons, a novel use of ANNs is proposed here to evaluate the adequacy of an input set of variables to classify the dependent variables.

### Searching automatically for discriminant characters: DTs vs. unsupervised DAs

Looking for a set of discriminant characters to distinguish taxonomic entities represents a primary objective in taxonomic studies [65]. In this study, DTs (11 variables selected, see Results) and unsupervised DAs (10 variables selected, see Results) were both appropriate to this aim. Despite the misclassification rates–suboptimal results using unsupervised DAs, as compared to DTs–the characters selected through DTs and unsupervised DAs are overall consistent with each other and the reference diagnostic characters (11 variables selected, see Results). That is, six variables coincided between guided DAs and DTs (DI, LT, LLM, STLM, STWM, and STLM/STWM), and a slightly different set of six features were shared between the two approaches that use DAs (DI, LT, LLM, STLM, STWM and WMPM).

The differences in the misclassification rate depend in part on the order of selection of diagnostic variables which eventually lead to the groupings. The variables which account for a greater amount of the variance were identified through unsupervised DAs. However, each step usually separated one or two species, but did not really split the dataset. This situation differed in the case of manual selection (guided DAs) or DTs, but neither the variable selection nor the order has one valid solution. Notably, the misclassification rate here was almost optimal using DTs. DTs achieved nearly the same results as guided DAs using a different combination of 11 variables from the initial set of 44. The similarity between these two techniques leads to some groupings that are equivalent (Group VII and C1) or quite comparable (Group I and B; Group III and C2).

DTs use specific classification rules allowing the direct and automatic creation of dichotomous keys to distinguish the different OTUs [101]. They also provide clear information on the importance of significant factors for prediction or classification [96]. In fact, DTs have been used in the pre-processing of data for the feature-selection step [98]. DTs (as data-mining techniques) can deal with sets having considerably high levels of incomplete data in several ways [102], but as mentioned above, they are not the most appropriate tools in the search of discriminant variables to classify species with low numbers of cases. By contrast, an important advantage of DAs is the ability to differentiate all the entities regardless of the number of observations. For particular scenarios involving endangered species or narrowly distributed taxa that, moreover, occur sympatrically with closely related species, DA may be the best choice [103,104]. In the present study, the species with low numbers of observations (e.g. LIN, TUR) were identified better by the combination of features implemented by DAs than by DTs.

For their part, DAs constitute an extraordinarily robust technique that in all cases should be considered in the search of morphological evidence for classification purposes (often in combination with molecular studies) when complete data sets are available [105–107]. Even with geometric morphometrics, multivariate analyses provide a good strategy for testing population differences [108]. With respect to the unsupervised DAs, the results would probably improve maintaining the sequential approach, but eliminating the restrictive rule of choosing the best variable in statistical terms and considering instead the set of the most useful variables with the help of graphic analysis (e.g. using box-plots).

### A “divide and conquer” strategy applied to the polyploid complex *V*. subsect *Pentasepalae*

The methodological approach followed in this study to search for diagnostic characters that could aid taxon classification is based on the “divide and conquer” principle. With this procedure, the most informative diagnostic characters were used to divide the initial dataset and progressively decrease its complexity. The methods that use recursive partitioning (i.e. splitting the initial task into various subtasks until they become simple enough to be easily solved) successfully address different kinds of intricate problems [109–111]. The combination of different techniques that are based on this principle (i.e. sequential DAs, DTs) seems to have been an excellent approach at least in the case of study. *Veronica* subsect. *Pentasepalae* is particularly challenging due not only to the high intraspecific morphological variability of some taxa, which makes species identification difficult, but also to the low number of populations known for several narrow endemics. However, this approach reduced the initial complexity, generating smaller subsets of data and avoiding the loss of information concerning the OTUs with low numbers of observations. It is noteworthy that in this case, the DTs, which implement a recursive partitioning method [73], provided satisfactory results. These recursive partitioning methodologies therefore seem to be reliable for assigning a population to taxa, either by conventional multivariate analysis techniques such as DAs, by implementing data-mining approaches, or by combining the two methods, which should not be considered mutually exclusive [89].

## Conclusions

In summary, the present study used the following workflow: 1) If possible, consider only individuals/populations which can be identified by other sources of information. Take detailed measurements corresponding to all the morphometric characters that *a priori* show variability (leaf features in this case, but any organ should be considered, and if that organ has a three-dimensional structure, geometric morphometrics should be considered as well). 2) Perform a PCoA or PCA (depending on the type of data) to verify whether there is enough variance present in the variables to explain the cases. 3) Implement a “divide and conquer” approach through the DT technique as a fast, easy, and effective solution in the search of diagnostic characters. In the case of species with low numbers of populations (or scarce data for any other reason), take advantage of the properties of the DAs to determine whether there is a sequence of characters that allows their classification aside from DTs and afterwards apply DTs without these entities. 4) Assess through ANNs the capacity of the variables to classify the taxa included in the final groups. Consider the variables selected as input layers and the taxa as output layers, divide the corresponding subset into several training/testing groups, and calculate the misclassification rate. If the rates show consistently high values or the different results are too unstable, the search of other characters would be recommended.

Establishing a general protocol based on this particular example seems of course too bold. However, these methodological guidelines may be of use to find robust morphological characters to differentiate among closely related taxa which have been taxonomically recognized as different entities based on multiple lines of evidence. Morphological data has its limitations in that it can disagree with phylogenetic data or can be misinterpreted due to homoplasy. Thus, gathering as many data as possible about species or infraspecific taxa (i.e. genetic, cytological, biogeographic, etc.) appears to be the most appropriate way to achieve classification. Integrative taxonomy appears to be the most suitable way to inspect biodiversity, the selection of the most appropriate combination of characters to identify each group of organisms is crucial, and morphological features should not be ruled out [65,112].

### Applications

As mentioned above, each group of organisms has its particularities (and hopefully its appropriate solutions). There are scenarios in which the extreme morphological and ecological variation among species [113] or the existence of cryptic taxa [114] has never allowed the identification of diagnostic phenotypic characters (by definition no morphological character would be found in the latter example) and cases in which genetic approaches show promising results [115,116].

In practical terms, conservationists cannot protect organisms that cannot be identified [117]. Adequate knowledge and description are needed to develop the necessary plans and mechanisms for species conservation [118,119]. For species complexes that are difficult to determine, it is recommended to perform careful morphometric studies on previously established taxa, which may allow finding robust characters in order to achieve proper identification. The adequate determination of endangered species and their distinctiveness with respect to their closest relatives is required, mainly when distribution areas are sympatric [120,121]. Studies based on the recursive partitioning or the “divide and conquer” principle are easily implementable to identification guides or even mobile apps (e.g., ArbolApp: http://www.arbolapp.es/; IPflanzen: http://www.ipflanzen.ch/; NatureGate: http://www.luontoportti.com/suomi/en/), which would increase the knowledge of species outside the academic sphere, thus facilitating their protection. The applications of these approaches may therefore facilitate the necessary dialogue with practitioners, communication that needs urgent improvement [122], even in order to avoid the imminent extinction of taxonomists, an additional endangered species [123].

## Supporting information

S1 Table Voucher information. Voucher information for the *Veronica* samples used in this study.
(DOCX)

S2 Table Guided DA information. Discriminant functions, standard coefficients and structure matrix tables for the sequential guided discriminant analysis.
(XLSX)

S3 Table Systematic DA information. Discriminant functions, standard coefficients and structure matrix tables for the systematic unsupervised discriminant analysis.
(XLSX)

S1 Fig Example of the architecture of an artificial neural network. (I) input layers = 15; (H) hidden layers = 1; number of neurons = 8; (O) output layers = 8. Output layers correspond to taxa (see Table 1 for abbreviations), input layers correspond to variables (see Table 2 for abbreviations). Positive and negative connections are represented by black and grey lines, respectively. Line width indicates the strength of the connection.
(TIF)

S2 Fig MCR vs. number of species. Misclassification rate in relation to the number of output layers (i.e., number of species and subspecies). Each point represents a different combination of randomly chosen OTUs.
(TIF)

S3 Fig Perfect tree. (TIF)

S1 File Box-plots corresponding to terminal groups (I to VIII) established through guided DAs. The black line in each box-plot indicates the threshold applied to perform the division.
(DOCX)

S2 File Box-plots showing the divisions performed with the most discriminant characters found through unsupervised DAs. The black line in each box-plot indicates the threshold that minimizes the misclassification rate.
(DOCX)
