# Supplementary material for: Divide and conquer! Data-mining tools and sequential multivariate analysis to search for diagnostic morphological characters within a plant polyploid complex (Veronica subsect. Pentasepalae, Plantaginaceae)
Source: PLoS One. 2018 Jun 29;13(6):e0199818. doi: 10.1371/journal.pone.0199818 (PMC6025878; doi:10.1371/journal.pone.0199818)
Supplement: S1 Table — Voucher information for the Veronica samples used in this study. (DOCX) [file pone.0199818.s001.docx]

| Accession number^a^  (Collectors^b^, date) | Species | Collection country and locality (Latitude, Longitude) | No. of individuals |
| --- | --- | --- | --- |
| BCF41230 (unknown collector, VI-1947) | *V. aragonensis* | Spain. Huesca, Guara.  (42.28142N, 00.14620W) | 3 |
| GDA18086 (Negrillo, 2-VII-1983) | *V. aragonensis* | Spain. Granada, La Sagra mountains.  (37.94617N, 02.56752W) | 3 |
| JACA256379 (Montserrat, 22-VII-1979) | *V. aragonensis* | Spain. Huesca, La Estiva. (42.47367N, 00.26289E) | 3 |
| JACA349179 (Villar, 7-VIII-1979) | *V. aragonensis* | Spain. Huesca, Yésero-Gavín. (42.67128N, 00.24202W) | 3 |
| JACA473871 (Montserrat, 15-VII-1971) | *V. aragonensis* | Spain. Huesca, Ordesa. (42.65647N, 00.01089E) | 3 |
| JACA52774 (Montserrat, 12-V-1974) | *V. aragonensis* | Spain. Huesca, Bentué de Rasal.  (42.33473N, 00.48740W) | 3 |
| JACA665787 (Sesé & Montserrat, 4-VII-1988) | *V. aragonensis* | Spain. Huesca, Montañeta de Gabas. (42.45913N, 00.46255E) | 3 |
| SALA153002 (Rojas-Andrés et al., 17-VII-2014) | *V. austriaca* subsp. *austriaca* | Slovakia. Spisšká Nová Ves. Letanovce. P.N. Slovensky raj. (48.95525N, 20.43775E) | 3 |
| SALA157057 (Martínez-Ortega et al., 16-VII-2014) | *V. austriaca* subsp. *austriaca* | Slovakia. Muráň, P.N. Muránska Planina.  (48.75112N, 20.04861E) | 3 |
| SALA157051 (Martínez-Ortega et al., 20-VII-2014) | *V. austriaca* subsp. *austriaca* | Rumania. Valea Lungă, Tauri monastery.  (46.14058N, 24.05728E) | 3 |
| SALA155836 (López-González et al., 18-VII-2014) | *V. austriaca* subsp. *austriaca* | Slovakia. Michalovce, Vinné, Viniansky Hrad.  (48.81709N, 21.94442E) | 3 |
| SALA157058 (López-González et al., 18-VII-2014) | *V. austriaca* subsp. *austriaca* | Slovakia. Černochov.  (48.43022N, 21.70586E) | 3 |
| B147196-138 (Bartha, 20-V-1929) | *V. austriaca* subsp. *dentata* | Hungary. Budapest, Ruppehegy mountain.  (47.46330N, 18.95022E) | 3 |
| B147196-23 (Bässler et al., 6-VI-1967) | *V. austriaca* subsp. *dentata* | Hungary. Bükk mountains in direction to Ómassa.  (48.10868N, 20.52962E) | 3 |
| B147196-6 (Preissmann, 19-V-1887) | *V. austriaca* subsp. *dentata* | Austria. Gösting in Grar. (47.10000N, 15.38333E) | 3 |
| BM67715 (Krebs, VII-1902) | *V. austriaca* subsp. *dentata* | Austria. Perchstoldorf.  (48.12044N, 16.26894E) | 3 |
| BM68162 (Sillinger, V-1930) | *V. austriaca* subsp. *dentata* | Czech Republic. Distr. Beroun, Doutnác hill near the village of Srbsko.  (49.95078N, 14.14976E) | 3 |
| BM68170 (Braun, date unknown) | *V. austriaca* subsp. *dentata* | Austria. Laxenburg.  (48.06865N, 16.35624E) | 3 |
| BM68180 (Sychowa & Jasiewicz, 17-V-1968) | *V. austriaca* subsp. *dentata* | Poland. Distr. Miechów. Kalina Mala.  (50.36648N, 20.11656E) | 3 |
| BM68606 (Weiss,16-VI-1883) | *V. austriaca* subsp. *dentata* | Germany. Bavaria, München, Garching.  (48.24883N, 11.65112E) | 3 |
| MA112519 (Skrenbergen, 18-V-1876) | *V. austriaca* subsp. *dentata* | Germany. Between Pebüsch and Calvarienberg.  (49.26333N, 9.148519E) | 3 |
| MA112520 (Laus, VI-1939) | *V. austriaca* subsp. *dentata* | Czech Republic. Olmütz. (49.57155N, 17.17745E) | 3 |
| SALA124613 (Martínez-Ortega, 15-V-2001) | *V. austriaca* subsp. *dentata* | Austria. Niederösterreich, NW Alpenostrand.  (48.04522N, 16.26287E) | 3 |
| SALA124612 (Martínez-Ortega, 3-VI-2001) | *V. austriaca* subsp. *dentata* | Slovakia. Bratislava, Thebener Kogel.  (48.18926N, 16.99543E) | 3 |
| SALA124579 (Albach, 12-VI-2001) | *V. austriaca* subsp. *jacquinii* | Bulgaria. Central Rhodopes, Asenograv.  (41.89500N, 29.92944E) | 3 |
| SALA124576 (Albach, 17-VI-2001) | *V. austriaca* subsp. *jacquinii* | Bulgaria. Vitosha mountain, mid-station of góndola. (42.56724N, 23.28578E) | 3 |
| SALA124581 (Albach, 17-VI-2001) | *V. austriaca* subsp. *jacquinii* | Bulgaria. Vitosha mountain, near point where road comes closest to góndola. (42.58861N, 23.31194E) | 3 |
| SALA124582 (Albach, 20-VI-2001) | *V. austriaca* subsp. *jacquinii* | Bulgaria. Vitosha mountain. Ljulimin, beneath ski slope. (42.56724N, 23.28578E) | 3 |
| SALA124583 (Albach, 20-VI-2001) | *V. austriaca* subsp. *jacquinii* | Bulgaria. Vitosha mountain. Ljulimin, beneath ski slope. (42.56724N, 23.28578E) | 3 |
| SALA124584 (Albach, 20-VI-2001) | *V. austriaca* subsp. *jacquinii* | Bulgaria. Eastern Stara mountain, 2 km south of Gindzi.  (42.43278N 25.64194E) | 3 |
| E32508 (Bujorean, 22-V-1922) | *V. austriaca* subsp. *jacquinii* | Romania. Banatus, distr. Caras-Severin.  (45.08333N 22.08333E) | 3 |
| K (Cook et al., 21-VII-1959) | *V. austriaca* subsp. *jacquinii* | Kosovo. Metohija, Rusolija Massif. (42.75056N 20.24528E) | 3 |
| K (Rechinger & Scheffer, 26-VII-1933) | *V. austriaca* subsp. *jacquinii* | Albania. Bertiscus, Greben mountain.  (42.55750N, 19.69556E) | 2 |
| SALA124610 (Martínez-Ortega, 18-V-2001) | *V. austriaca* subsp. *jacquinii* | Georgia. Kakheti, Shirakis vake, between Kvemo Bodve and Gamardzhveba.  (41.53528N, 45.90500E) | 3 |
| SALA124607 (Martínez-Ortega & Tribsch, 16-VI-2001) | *V. austriaca* subsp. *jacquinii* | Croatia. Velika Kapela, Gorska Kotar, P. N. Risnjak.  (45.42083N, 14.56306E) | 2 |
| SALA124604 (Martínez-Ortega & Tribsch, 16-VI-2001) | *V. austriaca* subsp. *jacquinii* | Croatia. Velika Kapela, Gornje Jelenje, in the crossroad to Crikvanica.  (45.36583N, 14.61639E) | 3 |
| SALA124605 (Martínez-Ortega & Tribsch, 16-VI-2001) | *V. austriaca* subsp. *jacquinii* | Croatia. Between Mrkopajl and Begovo Razdolje.  (45.30361N, 14.89750E) | 3 |
| SALA124603 (Martínez-Ortega & Tribsch, 17-VI-2001) | *V. austriaca* subsp. *jacquinii* | Croatia. Opatija, road to Ljubljana between Rupa and Lipa. (45.47833N, 14.28639E) | 3 |
| SALA124609 (Martínez-Ortega & Tribsch, 17-VI-2001) | *V. austriaca* subsp. *jacquinii* | Croatia. Licausko, Polje. (45.13667N, 14.64222E) | 3 |
| SALA124606 (Martínez-Ortega, 18-VI-2001) | *V. austriaca* subsp. *jacquinii* | Croatia. Velebit, Mali Alan pass, between Obrovac and Sveti Rok. (44.20056N, 15.68222E) | 3 |
| SALA124608 (Martínez-Ortega et al., 4-VII-2002) | *V. austriaca* subsp. *jacquinii* | Slovakia. Cernochov, Vychodoslovenska lowlands. (48.43750N, 21.73833E) | 3 |
| SALA124564 (Schneeweiss et al., 21-VI-2001) | *V. austriaca* subsp. *jacquinii* | Croatia. Zadar, Velebit, N. P. Paklenica.  (44.34040N, 15.47480E) | 3 |
| RNG (Stamatiadou, 13-VI-1971) | *V. austriaca* subsp. *jacquinii* | Greece. Sterea Ellas, distr. Fthiotis, Othris mountain. SE side of Jerakovouni.  (39.03333N, 22.61667E) | 3 |
| SALA149288 (Rojas-Andrés et al., 26-VII-2010) | *V. crinita* | Serbia. Between Zlot and Brestovac.  (44.02367N, 21.99328E) | 3 |
| SALA149289 (Rojas-Andrés et al., 23-VI-2009) | *V. crinita* | Turkey. Dereköy, road to Geçitagzi. (41.93761N, 27.35306E) | 3 |
| SALA149038 (Santos et al., 18-VI-2009) | *V. crinita* | Bulgaria. Around Popovitsa. (42.12294N, 25.07567E) | 3 |
| SALA149290 (Santos et al., 21-VI-2009) | *V. crinita* | Bulgaria. Around Veliki Preslav. (43.13978N, 26.80806E) | 3 |
| SALA149037 (Santos et al., 22-VI-2009) | *V. crinita* | Bulgaria. Varna, between Vinitsa and Aladza monastery. (43.25097N, 28.00242E) | 3 |
| SALA149244 (Frajman & Schönswetter, 23-V-2009) | *V. crinita* | Bosnia-Herzegovina. Ravan mountain. Summit of Mt. Tajan. (44.28861N, 18.19028E) | 3 |
| PRC (Fiala, 1892) | *V. crinita* | Bosnia-Herzegovina. Klek mountain close to Foça.  (45.26083N, 15.14528E) | 3 |
| PRC (Fiala, 1892) | *V. crinita* | Bosnia-Herzegovina. Klek mountain. (45.26083N, 15.14528E) | 1 |
| PRC455004 (Beck, date unknown) | *V. crinita* | Bosnia-Herzegovina. Stražica in Vranica mountains. (43.95417N, 17.7711E) | 3 |
| SALA157013 (Rojas-Andrés et al., 9-VII-2014) | *V. kindlii* | Greece. Above Boras ski resort. (40.91902N, 21.80960E) | 3 |
| SALA149277 (Martínez-Ortega et al.,19-VII-2010) | *V. kindlii* | Montenegro. Cakor, next to Kosovo border.  (42.66072N, 19.99247E) | 3 |
| SALA149278 (Martínez-Ortega et al., 25-VII-2010) | *V. kindlii* | FYROM. Gevgelija, in the way to Kozuf ski resort.  (41.20006N, 22.24369E) | 3 |
| SALA157011 (Martínez-Ortega et al., 8-VII-2014) | *V. kindlii* | FYROM. P. N. Pelister, Pelister mountain.  (40.99456N, 21.17847E) | 3 |
| SALA157012 (Martínez-Ortega et al., 8-VII-2014) | *V. kindlii* | FYROM. P. N. Pelister, Pelister mountain.  (40.99456N, 21.17847E) | 3 |
| SALA149346 (Santos et al., 25-VI-2009) | *V. kindlii* | Greece. Vermion mountain. (40.53606N, 22.02403E) | 3 |
| SALA149279 (Santos et al., 27-VI-2009) | *V. kindlii* | Greece. In the ascent to Kaïmaktsalán mountain. (40.90708N, 21.82561E) | 3 |
| SALA149282 (Santos et al., 27-VI-2009) | *V. kindlii* | Greece. In the ascent to Kaïmaktsalán mountain. (40.90708N, 21.82561E) | 3 |
| DR041947 (Behr, 1937) | *V. linearis* | FYROM. Sivec at Prilep. (41.40944N, 21.59194E) | 3 |
| SALA153001 (Martínez-Ortega et al., 11-VII-2014) | *V. linearis* | FYROM. Between Modrište and Zdunje, next to Jezero Kozjak. (41.72449N, 21.19320E) | 3 |
| B147196-192 (Maly, VI-1909) | *V. orbiculata* | Bosnia-Herzegovina. Sarajevo, below Mrkovic.  (43.84864N, 18.35644E) | 3 |
| BM68701 (Maly, 26-V-1907) | *V. orbiculata* | Bosnia-Herzegovina. Sarajevo. (43.84864N, 18.35644E) | 3 |
| SALA149336 (Rojas-Andrés et al., 11-VII-2010) | *V. orbiculata* | Bosnia-Herzegovina. Mostar, in the ascent to Hum mountain. (43.32728N, 17.79939E) | 3 |
| SALA149294 (Rojas-Andrés et al., 14-VII-2010) | *V. orbiculata* | Croatia. Peljesak Peninsula, between Trstenik and Pijavicino. (42.93728N, 17.37764E) | 3 |
| K (Raap, 1895) | *V. orbiculata* | Bosnia-Herzegovina. Mostar. (43.34333N, 17.80806E) | 3 |
| SALA124585 (Martínez-Ortega, 19-VI-2001) | *V. orbiculata* | Croatia. Brela, between Omis and Makarska.  (43.33000N, 17.07000E) | 3 |
| SALA124587 (Martínez-Ortega, 20-VI-2001) | *V. orbiculata* | Croatia. Makarska, between Tucepi and G. Igrane.  (43.23222N, 17.14361E) | 3 |
| SALA124586 (Martínez-Ortega & Solic, 20-VI-2001) | *V. orbiculata* | Croatia. Makarska, Osejaba. (43.29694N, 17.01778E) | 3 |
| SALA149337 (Martínez-Ortega et al., 10-VI-2010) | *V. orbiculata* | Croatia. Brela, crossroad towards Gornja Brela.  (43.40383N, 16.89364E) | 3 |
| SALA149295 (Martínez-Ortega et al., 10-VI-2010) | *V. orbiculata* | Croatia. Prapatnice, in the ascent to Matokit.  (43.22114N, 17.35972E) | 3 |
| RNG (Halliday, 15-IV-1972) | *V. orbiculata* | Montenegro. Budva, behind Podostrog monastery.  (42.28639N, 18.84000E) | 3 |
| SALA149297 (Rico et al., 6-VII-2002) | *V. orsiniana* | Italy. P. N. d’Abruzzo, La Majella. (42.07556N, 14.09694E) | 3 |
| SALA149298 (Rico et al., 6-VII-2002) | *V. orsiniana* | Italy. P. N. d’Abruzzo, La Majella. (42.07556N, 14.09694E) | 3 |
| CAME (Conti, 30-V-1995) | *V. orsiniana* | Italy. P. N. d'Abruzzo,Colle Biferno. (41.93794N, 13.73200E) | 3 |
| SALA110621 (Delgado et al., 11-V-1999) | *V. orsiniana* | Spain. Riudellots de la Selva. (41.89500N, 02.80694E) | 3 |
| SALA110622 (Delgado et al., 9-V-1999) | *V. orsiniana* | Spain. Malla, Alto del Closcar. (41.88152N, 02.21663E) | 3 |
| SALA110626 (Delgado et al., 11-V-1999) | *V. orsiniana* | Spain. Montserrat, Ermita de San Miguel.  (41.58667N, 01.84139E) | 3 |
| MA180274 (Gavelle, 28-VI-1957) | *V. orsiniana* | France. Nice, Gros mountain. (43.72916N, 07.29851E) | 3 |
| MA425880 (Tallon, 14-V-1931) | *V. orsiniana* | France. Gard, Pont du Gard. (43.94683N, 04.53606E) | 3 |
| SALA93492 (Martínez-Ortega & Martín Ballesteros, 20-VI-1996) | *V. orsiniana* | Spain. Huesca, Sobrarbe, Chisagüés valley.  (42.66489N, 00.18595W) | 3 |
| SALA93483 (Martínez-Ortega & Delgado, 8-VII-1997) | *V. orsiniana* | Spain. Huesca, Borau, As Blancas. (42.67833N, 00.570000W) | 3 |
| SALA93482 (Martínez-Ortega & Delgado, 10-VII-1997) | *V. orsiniana* | Spain. From Torla to Bujaruelo, Los Navarros bridge.  (42.65333N, 00.102780W) | 3 |
| SALA110623 (Martínez-Ortega et al., 10-V-1999) | *V. orsiniana* | Spain. Barcelona, Tona, Virgen de Lourdes sanctuary.  (41.84549N, 02.217070W) | 3 |
| SALA124591 (Martínez-Ortega et al., 10-V-1999) | *V. orsiniana* | Spain. Santa Cecilia de Voltregá, km 3 in the road to Manlleu.  (41.98950N, 02.203240E) | 3 |
| SALA110624 (Martínez-Ortega et al., 10-V-1999) | *V. orsiniana* | Spain. Road from Vic to Villadrau. (41.84639N, 02.36160E) | 3 |
| SALA110625 (Martínez-Ortega et al., 12-V-1999) | *V. orsiniana* | Spain. El Miracle, road Cardona-El Miracle.  (41.910640N, 01.51697E) | 3 |
| SALA124590 (Martínez-Ortega & Delgado, 11-VI-1999) | *V. orsiniana* | Spain. Luesia, Luesia mountains. (42.40702N, 00.99497W) | 3 |
| SALA110619 (Martínez-Ortega & Delgado, 13-VI-1999) | *V. orsiniana* | Spain. Loarre, Loarre mountains, signal repeater.  (42.33832N, 00.69371W) | 3 |
| SALA124588 (Martínez-Ortega & Delgado, 2-VIII-1999) | *V. orsiniana* | Spain. Huesca, Puértolas, Castillo Mayor.  (42.57583N, 00.12222W) | 3 |
| SALA93486 (de Retz, 1-VI-1990) | *V. orsiniana* | France. Dép. Alpes-Maritimes, Caussols.  (43.73528N, 06.92836E) | 3 |
| SALA72239 (Biondi & Amor, 29-V-1992) | *V. orsiniana* | Italy. Umbria, Perugia, Spello, Monte Subasio.  (43.05000N, 12.66667E) | 3 |
| VAB932949 (Fabregat & Lopez, 19-VI-1993) | *V. orsiniana* | Spain. Teruel, Iglesuela del Cid. (40.48312N 00.31938W) | 3 |
| VAB933759 (Fabregat & Lopez, 19-VI-1993) | *V. orsiniana* | Spain. Castellón, Villafranca. (40.42853N, 00.25771W) | 3 |
| VAB944259 (Mercadal, 12-VII-1993) | *V. orsiniana* | Spain. Teruel, Cantavieja. (40.53295N, 00.48806W) | 3 |
| VAB947095 (Fabregat & López, 8-VI-1991) | *V. orsiniana* | Spain. Castellón, Villafranca, La Moleta.  (40.42196N, 00.33607W) | 3 |
| B (Damboldt, 30-V-1970) | *V. prostrata* | Germany. Berlin, Glienicker Volkspark meadow.  (52.44250N 13.58222W) | 3 |
| BC661136 (Negri, VII-1877) | *V. prostrata* | Italia. Casale Monferrato hill. (45.13333N, 08.45694E) | 3 |
| BM68672 (Garllorph, V-1905) | *V. prostrata* | Germany. Potsdam. (52.39667N, 13.05836E) | 3 |
| BM68674 (Mayer, 26-IV-1967) | *V. prostrata* | Serbia. Banat, Dliblatska peščara, Devojacki bunar.  (44.89874N, 21.12988E) | 3 |
| BM68702 (Schneider, 16-V-1907) | *V. prostrata* | Bulgaria. Varna.  (43.21667N, 27.91667E) | 3 |
| BM68710 (Filarszky & Kümmerle, 9-V-1916,) | *V. prostrata* | Slovakia. Comit Szepes, Smižany. (48.95525N, 20.51774E) | 3 |
| BM68720 (Toma, 10-V-1962) | *V. prostrata* | Romania. Iasi Breazu.  (47.21667N, 27.51667E) | 2 |
| BM68839 (Lacaita, 6-V-1906) | *V. prostrata* | Italy. Toscana, Monte di prato. (44.05894N, 10.61664E) | 3 |
| SALA124616 (Martínez-Ortega, 28-V-2001) | *V. prostrata* | Austria. Leithagebirge, Donnerskirchen.  (47.93321N, 16.66752E) | 3 |
| SALA124614 (Martínez-Ortega, 3-VI-2001) | *V. prostrata* | Austria. Hainburg, Braunsberg. (48.15406N, 16.95782E) | 2 |
| SALA124619 (Martínez-Ortega, 10-VI-2001) | *V. prostrata* | Czech Republic. Srbsko. (49.95219N, 14.14736E) | 3 |
| SALA124615 (Martínez-Ortega, 11-VI-2001) | *V. prostrata* | Austria. Falkenstein.  (48.72445N, 16.57919E) | 3 |
| SALA124617 (Martínez-Ortega, 24-VI-2001) | *V. prostrata* | Czech Republic. Between Mikulov and Klentnice.  (48.82603N, 16.64077E) | 3 |
| SALA124620 (Martínez-Ortega et al., 30-VI-2000) | *V. prostrata* | Romania. Fanatele Clujului, 8 Km from Cluj-Napoca.  (46.85247N, 23.61688E) | 3 |
| SEV16475 (Bührer, 24-V-1950) | *V. prostrata* | Switzerland. Kt. Wallis, Gemeinde Orsières, Westhaang.  (46.03850N, 07.15628E) | 2 |
| SALA149321 (Rojas-Andrés et al., 13-VI-2009) | *V. rhodopea* | Bulgaria. Pazardzhik, Belmeken.  (42.17653N, 23.80769E) | 3 |
| C (Bondev, 1955) | *V. rhodopea* | Bulgaria. Rila mountain.  (42.18333N, 23.74361E) | 2 |
| PR806998 (Klásterský & Deyl, date unknown) | *V. rhodopea* | Bulgaria. Kara Balkan mountains. (41.58528N, 24.69194E) | 3 |
| B100217752 (Markgraf & Markgraf, 1973) | *V. rhodopea* | Bulgaria. Beglika southwards Pestera.  (41.81512N, 24.12809E) | 3 |
| PRC455001 (Mrkvicka, 1916) | *V. rhodopea* | Bulgaria. Dobro Pole.  (N/A) | 3 |
| BM67493 (Ibrahim, 5-VI-1884) | *V. rosea* | Morocco. Lalla-Aziza.  (31.08505N, 08.70431W) | 3 |
| BM67502 (Davis, 21-VI-1975) | *V. rosea* | Algeria. Djurdjura, between Tizi-N’Kouilal pass and Tikdja. (36.47669N, 04.23206E) | 3 |
| BM67505 (Faure, 29-VI-1930) | *V. rosea* | Algeria. Bossuet.  (34.66213N, 00.62089W) | 3 |
| BM67508 (Harley, 5-VII-1966) | *V. rosea* | Morocco. Beni Mellal, below summit of Irhil Ouaougoulzate.  (31.65000N, 06.26667W) | 3 |
| BM67522 (Font Quer, 22 VII-1929) | *V. rosea* | Morocco. Djebel Lakraa.  (35.13658N, 05.13711W) | 3 |
| E32376 (Alexander & Kupicha, 26-V-1972) | *V. rosea* | Morocco. Ketama-El Hoceima. (34.99332N, 04.21485W) | 2 |
| G8263-1733 (Bourgeau, 21-V-1856) | *V. rosea* | Algeria. Wilaya Oran, Ghar Rouban mountain.  (34.58590N, 01.78783W) | 3 |
| G8263-1736 (Podlech, 7-VI-1984) | *V. rosea* | Algeria. Wilaya Batna, Awras region, Djebel Chélia.  (35.49916N, 06.16661E) | 3 |
| G8263-1746 ( Wilczek et al, 20-IV-1928) | *V. rosea* | Morocco. Beni Suassene, refuge Zegzel. (34.83501N, 02.353421W) | 3 |
| G8263-1767 (Charpin et al., 31-V-1980) | *V. rosea* | Morocco. Ouarzazate, next to Tizi n’Melloul. (30.78333N, 07.60000W) | 3 |
| GDA27834 (Font Quer, 29-VI-1927) | *V. rosea* | Morocco. El Ferrah, Beni Hadifa. (35.02060N, 04.14370W) | 3 |
| K (Wilczek et al., 20-IV-1928) | *V. rosea* | Morocco. Beni Suassene, Tamcojoutan mountain.  (34.85001N, 02.34996W) | 3 |
| MA302831 (Blanché et al., 1-VI-1985) | *V. rosea* | Morocco. Ouarzazate, Tizi n’Ouaro. (31.96666N, 05.63333W) | 3 |
| MA429804 (Sennen, date unknown) | *V. rosea* | Morocco. Riff.  (34.99269N, 04.00250W) | 2 |
| BC654127 (Don de Fritz, 18-VII-1872) | *V. satureiifolia* | Switzerland. Jura Neuchâteloise, Vallon de la Brèvine.  (46.98685N, 06.60921E) | 3 |
| BC832014 (Despaty, 20-V-1917) | *V. satureiifolia* | France. Seine et Oise, Champareil. (49.27036N, 02.50791E) | 3 |
| G8263/1680 (Beckerer, 4-VII-1938) | *V. satureiifolia* | Austria. Valais, colline northwards d'Orsiéres.  (46.17798N, 07.57521E) | 2 |
| G8263/1718 (Vautier, 22-V-1954) | *V. satureiifolia* | Switzerland. Dep. Lozère Aveu Armand, Causse méjean. (44.57987N, 03.71332E) | 2 |
| JACA10062571 (Villar, 2-VI-1971) | *V. satureiifolia* | Spain. Navarra, Isaba, Belagua. (42.89472N, 00.78111W) | 3 |
| JACA680871 (Montserrat & Villar, 2-IX-1971) | *V. satureiifolia* | Spain. Huesca, Hecho, Alanos, La Renclusa.  (42.81950N, 00.83000W) | 3 |
| JACA71965 (Montserrat, date unknown) | *V. satureiifolia* | Spain. Navarra, Belagua, Roncal. (42.94508N, 00.83041W) | 3 |
| MA185334 (Paul, 3-VI-1933) | *V. satureiifolia* | Germany. Schwaben, Marienhöhe to Nördlingen.  (48.83791N, 10.49653E) | 3 |
| SALA124594 (Martínez-Ortega, 9-V-2001) | *V. satureiifolia* | Germany. Baden Württhemberg, Schwäbische.  (48.83556N, 10.37028E) | 2 |
| SALA124595 (Martínez-Ortega, 9-V-2001) | *V. satureiifolia* | Germany. Baden Württhemberg, Schwäbische.  (48.80917N, 10.40528E) | 3 |
| SALA93470 (Martínez-Ortega & Delgado, 7-VII-1997) | *V. satureiifolia* | Spain. Huesca, Aragüés del Puerto, Llanos de Lizara.  (42.76944N, 00.63361W) | 3 |
| SALA93472 (Martínez-Ortega & Delgado, 7-VII-1997) | *V. satureiifolia* | Spain. Huesca, Aragües del Puerto, Collado de Mesola.  (42.74028N, 00.61278W) | 3 |
| SALA93471 (Martínez Ortega & Delgado, 10-VII-1997) | *V. satureiifolia* | Spain. Huesca, Hoz de Jaca, in the ascending to El Mandilar. (42.69333N, 00.28083W) | 3 |
| SALA124592 (Martínez Ortega & Delgado, 5-VIII-1999) | *V. satureiifolia* | Spain. Huesca, Aragües del Puerto, Pico Cucuruzuelo.  (42.72472N 00.62361W) | 3 |
| SALA124593 (Martínez Ortega & Delgado, 6-VIII-1999) | *V. satureiifolia* | Spain. Huesca, Ansó, Paso del Onso.  (42.89472N, 00.78111W) | 3 |
| FCO04428 (Navarro Andrés, 15-VII-1973) | *V. senneni* | Spain. Asturias, Valdemurrio water reservoir.  (43.19836N, 06.01512W) | 2 |
| SALA110642 (Delgado & Rico, 7-VI-2001) | *V. senneni* | Spain. León, Valdepiélago, Valdorria.  (42.88517N, 05.42452W) | 3 |
| SALA110637 (Delgado & Rico, 7-VI-2001) | *V. senneni* | Spain. León, Valdelugueros, Las Majadas.  (42.92141N, 05.41369W) | 3 |
| SALA110634 (Delgado & Rico, 7-VI-2001) | *V. senneni* | Spain. León, Riaño.  (42.93779N, 05.03442W) | 3 |
| MA532538 (Gil de Zúñiga & Alejandre, 23-VI-1991) | *V. senneni* | Spain. La Rioja, Pedroso, Camero Nuevo mountains.  (42.28194N, 02.66599W) | 3 |
| SALA93480 (Martínez-Ortega, 2-V-1997) | *V. senneni* | Spain. Álava, Salinas de Añana (Gesaltza Añana), trail from Sobrón to Rastrilla hill.  (42.787872N, 3.092367W) | 3 |
| SALA93495 (Martínez-Ortega & Martín Ballesteros, 23-VI-1996) | *V. senneni* | Spain. Alava, Salinas de Añana (Gesaltza Añana). (42.77571N, 03.12224W) | 3 |
| SALA93479 (Martínez-Ortega, 2-V-1997) | *V. senneni* | Spain. Alava, Valdegobía, Villamardones.  (42.86694N, 03.25377W) | 3 |
| SALA93478 (Martínez-Ortega, 3-V-1997) | *V. senneni* | Spain. Alava, Pipaón.  (42.61309N, 02.63424W) | 3 |
| SALA93477 (Martínez-Ortega, 26-IV-1999) | *V. senneni* | Spain. Cantabria, Castro Urdiales, Oriñón.  (43.39664N, 03.32104W) | 3 |
| SALA93475 (Martínez-Ortega, 26-IV-1999) | *V. senneni* | Spain. Cantabria, Laredo, El Puntal. (43.43220N, 03.45714W) | 3 |
| SALA110638 (Martínez-Ortega et al., 18-VI-1999) | *V. senneni* | Spain. Navarra, Aralar mountains. (43.00580N, 02.03073W) | 2 |
| SALA93485 (Uribe-Echebarría, 21-VI-1991) | *V. senneni* | Spain. Alava, Valdegobía, Lalastra. (42.867214N, 03.20272W) | 2 |
| SALA93487 (Martínez & Morante, 17-VI-1984) | *V. senneni* | Spain. Alava, Arlucena. (42.72678N, 02.54325W) | 2 |
| SEST38691 (Patino & Valencia, 27-V-1991) | *V. senneni* | Spain. Burgos, Junta de Trasloma.  (43.03776N, 03.38948W) | 3 |
| GDA26404 (Torres et al., 9-VI-1983) | *V. tenuifolia* subsp. *fontqueri* | Spain. Granada, Baza mountains. (37.37903N, 02.84187W) | 3 |
| MA389465 (Fernández Casas, 19-VII-1974) | *V. tenuifolia* subsp. *fontqueri* | Spain. Granada, Baza mountains, Los Tejos pass.  (37.37903N, 02.84187W) | 2 |
| MGC36827 (Cabezudo et al., 4-VII-1991) | *V. tenuifolia subsp. fontqueri* | Spain. Málaga, Yunquera, las Nieves mountains, Peña de los Enamorados.  (36.69500N, 05.01130W) | 3 |
| MGC46659 (Cabezudo & Martínez-Ortega, 9-VI-1998) | *V. tenuifolia* subsp. *fontqueri* | Spain. Málaga, Ronda, las Nieves mountains, Los Quejigales. (36.68611N, 05.03028W) | 3 |
| SALA95041 (Martínez-Ortega, 11-VI-1998) | *V. tenuifolia* subsp. *fontqueri* | Spain. Almería, Fondón, Gador mountains, El Boliche plain. (36.91023N, 02.79794W) | 3 |
| B147196 (Preissmann, 19-V-1887) | *V. tenuifolia* subsp. *javalambrensis* | Spain. Teruel, Tramacastilla mountains.  (40.42611N, 01.60000W) | 3 |
| E32503 (Brummit et al., date unknown) | *V. tenuifolia* subsp. *javalambrensis* | Spain. Cuenca, Valdeminguete mountains.  (40.34586N, 01.77017W) | 2 |
| JACA463686 (Montserrat & Montserrat, 6-VII-1986) | *V. tenuifolia* subsp. *javalambrensis* | Spain. Lérida, Puigcerda-Martinet. (42.35956N, 01.69584E) | 3 |
| MA339646 (Fz. de Betoño & Alejandre, 6-VI-1985) | *V. tenuifolia* subsp. *javalambrensis* | Spain. Burgos, Sargentes de la Lora.  (42.76917N, 03.87278W) | 3 |
| MA468044 (Gil de Zúñiga & Alejandre, 11-VI-1988) | *V. tenuifolia* subsp. *javalambrensis* | Spain. Soria, Villaciervos, Cabrejas mountains.  (41.78333N, 02.80000W) | 3 |
| MA532710 (Alejandre, 30-V-1990) | *V. tenuifolia* subsp. *javalambrensis* | Spain. Palencia, Cervera de Pisuerga, Peña de Santa Lucía. (42.91590N, 04.64302W) | 3 |
| SALA93463 (Martínez-Ortega, 14-V-1996) | *V. tenuifolia* subsp. *javalambrensis* | Spain. Salamanca, La Mata de la Armuña.  (41.03317N, 05.68832W) | 3 |
| SALA1411 (Rivas & Fernandez Galiano, 18-V-1952) | *V. tenuifolia* subsp. *javalambrensis* | Spain. Madrid, Cabrizos de Chozas. (40.41045N, 03.70175W) | 2 |
| SALA49322 (Casaseca et al., 11-VI-1990) | *V. tenuifolia* subsp. *javalambrensis* | Spain. Zamora, Abezames. (41.62642N, 05.42577W) | 3 |
| SALA93456 (Martínez-Ortega, 12-VII-1996) | *V. tenuifolia* subsp. *javalambrensis* | Spain. Cantabria, Camaleño, Mediana mountains.  (43.07727N, 04.76881W) | 3 |
| SALA93468 (Martínez-Ortega, 7-VI-1996) | *V. tenuifolia* subsp. *javalambrensis* | Spain. Segovia, Navares de las Cuevas, Pradales mountains. (41.45927N, 03.73041W) | 3 |
| VIT37034 (Alejandre et al., 20-VI-1986) | *V. tenuifolia* subsp. *javalambrensis* | Spain. Guadalajara, from Buenafuente to Huertahernan. (40.81410N, 02.27383W) | 3 |
| BC113724 (Font Quer, 9-V-1926) | *V. tenuifolia* subsp. *tenuifolia* | Spain. Cataluña, close to Almacelles.  (41.73216N, 0.437220E) | 3 |
| JACA61188 (Gómez & Aseginolaza, 16-V-1980) | *V. tenuifolia* subsp. *tenuifolia* | Spain. Zaragoza, Bardenas Reales, next to Tres Mugas.  (42.29040N, 01.39557W) | 3 |
| JACA77571 (Montserrat, 4-V-1971) | *V. tenuifolia* subsp. *tenuifolia* | Spain. Lérida, Organya. (42.19681N, 01.31644E) | 3 |
| JACA9857 (Montserrat, 29-IV-1957) | *V. tenuifolia* subsp. *tenuifolia* | Spain. Huesca, Mediano. (42.31899N, 00.19680E) | 2 |
| MAF108794 (Velasco, 10-VI-1977) | *V. tenuifolia* subsp. *tenuifolia* | Spain. Toledo, Montes de Toledo, Rebollarejo mountains. (39.45444N, 03.96480W) | 3 |
| SALA95040 (Martínez-Ortega, 7-VI-1998) | *V. tenuifolia* subsp. *tenuifolia* | Spain. Navarra, Cáseda. (42.38666N, 01.42078W) | 3 |
| RNG (Heywood, 16-V-1971) | *V. tenuifolia* subsp. *tenuifolia* | Spain. Barcelona, La Panadella. (41.42293N, 01.39684E) | 3 |
| K (Reverchon, VI-1891) | *V. tenuifolia* subsp. *tenuifolia* | Spain. Valencia, Segorbe mountains, Montemalo.  (39.81436N, 00.54648W) | 3 |
| SALA33052 (Peris & Stübing, 20-VI-1984) | *V. tenuifolia* subsp. *tenuifolia* | Spain. Valencia, Requena, Pico Tejo.  (39.51882N, 00.98775W) | 3 |
| VAB80719 (Mansanet & Mateo, VI-1980) | *V. tenuifolia* subsp. *tenuifolia* | Spain. Castellón, Forcall. (40.64542N, 00.19992W) | 3 |
| SALA149270 (Herrero, 1-VII-2007) | *V. teucrioides* | Greece. Kozani, Askio, Siniatsikon mountain.  (40.40333N, 21.53722E) | 3 |
| SALA149329 (Rojas-Andrés et al., 23-VII-2010) | *V. teucrioides* | FYROM. P.N. Mavrovo, Bistra mountain.  (41.64972N, 20.71075E) | 3 |
| SALA149330 (Rojas-Andrés et al., 26-VI-2009 | *V. teucrioides* | Greece. Olimpo mountain.  (40.03872N, 22.33369E) | 3 |
| SALA124600 (Schneeweiss et al., 23-VII-2001) | *V. teucrium* | Italy. Aosta, Valle di la Thuile. (45.70889N, 06.94056E) | 3 |
| BM68135 (Filarszky , 28-VI-1814) | *V. teucrium* | Slovakia. Szepes, Igló.  (47.45281N, 21.61058E) | 3 |
| BM68159 (Sag, 2-X-1875) | *V. teucrium* | Hungary. Visegrad.  (47.80000N, 18.98333E) | 2 |
| BM68550 (Palkowa & Necka, 27-VI-1974) | *V. teucrium* | Poland. Distr. Chrzanów. (50.13317N, 19.40050E) | 3 |
| SALA124597 (Albach, 15-VI-2001) | *V. teucrium* | Bulgaria. Znepole region, Tran. (42.83778N, 22.69083E) | 3 |
| SALA124578 (Albach, 17-VI-2001) | *V. teucrium* | Bulgaria. Vitosa mountain, Simeonovo, base station. (42.61556N, 23.34750E) | 3 |
| WU (Scheneider, 14-VI-1937) | *V. teucrium* | Austria. Kärnten, Schneeweiss. (46.70907N, 14.17363E) | 3 |
| MA112464 (unknown collector, 20-V-1929) | *V. teucrium* | Germany. Palatinat.  (49.19198N, 08.11498E) | 3 |
| MA185323 (Putchler, 8-VI-1918) | *V. teucrium* | Germany. Oberfranken Stadtsteinach. (50.16039N, 11.50514E) | 3 |
| MA333222 (Andrsovszky, 4-VI-1916) | *V. teucrium* | Hungary. Szent, Stara-Vod valley. (47.70529N, 19.04510E) | 3 |
| SALA93469 (Castroviejo et al., 17-VII-1990) | *V. teucrium* | Switzerland. Grisons, Engadina Bassa, Guarda.  (46.77585N, 10.15289E) | 3 |
| SALA124599 (Martínez-Ortega, 3-VI-2001) | *V. teucrium* | Slovakia. Bratislava, Thebener Kogel.  (48.19250N, 16.99833E) | 3 |
| SALA124602 (Martínez-Ortega & Fischer, 28-VI-2001) | *V. teucrium* | Austria. Niederösterreich between Pottenstein and Weissenbach. (47.96944N, 16.06333E) | 3 |
| SALA124598 (Martínez-Ortega et al., 3-VII-2002) | *V. teucrium* | Slovakia. Dvorníky-Vceláre, Zádiel. (48.61278N, 20.84222E) | 3 |
| SALA124562 (Schanzer & Majorov, 7-VII-2001) | *V. teucrium* | Russia. Tula, S valley of Krasivaya, Mecha Riv.  (53.23750N, 38.35944E) | 2 |
| SALA149331 (Rojas-Andrés et al., 23-VI-2009) | *V. turrilliana* | Turkey. Dereköy to Armagan. (41.90808N, 27.38706E) | 3 |
| SALA149333 (Rojas-Andrés et al., 23-VI-2009) | *V. turrilliana* | Turkey. 6 Km. from Vize, in the way to Kömürköy-Alkpinar.  (41.59472N, 27.82417E) | 3 |
| SALA149334 (Santos et al., 22-VI-2009) | *V. turrilliana* | Bulgaria. 15 Km northwards Malko Turnovo, western from the bridge upon Veleka river.  (42.08506N, 27.42903E) | 3 |

^a^ Herbarium and herbarium codes when available

^b^ When there are more than two collectors only the first one is displayed followed by et al.
