# Supplementary figures and images for: Divide and conquer! Data-mining tools and sequential multivariate analysis to search for diagnostic morphological characters within a plant polyploid complex (Veronica subsect. Pentasepalae, Plantaginaceae)
Source: PLoS One. 2018 Jun 29;13(6):e0199818. doi: 10.1371/journal.pone.0199818 (PMC6025878; doi:10.1371/journal.pone.0199818)

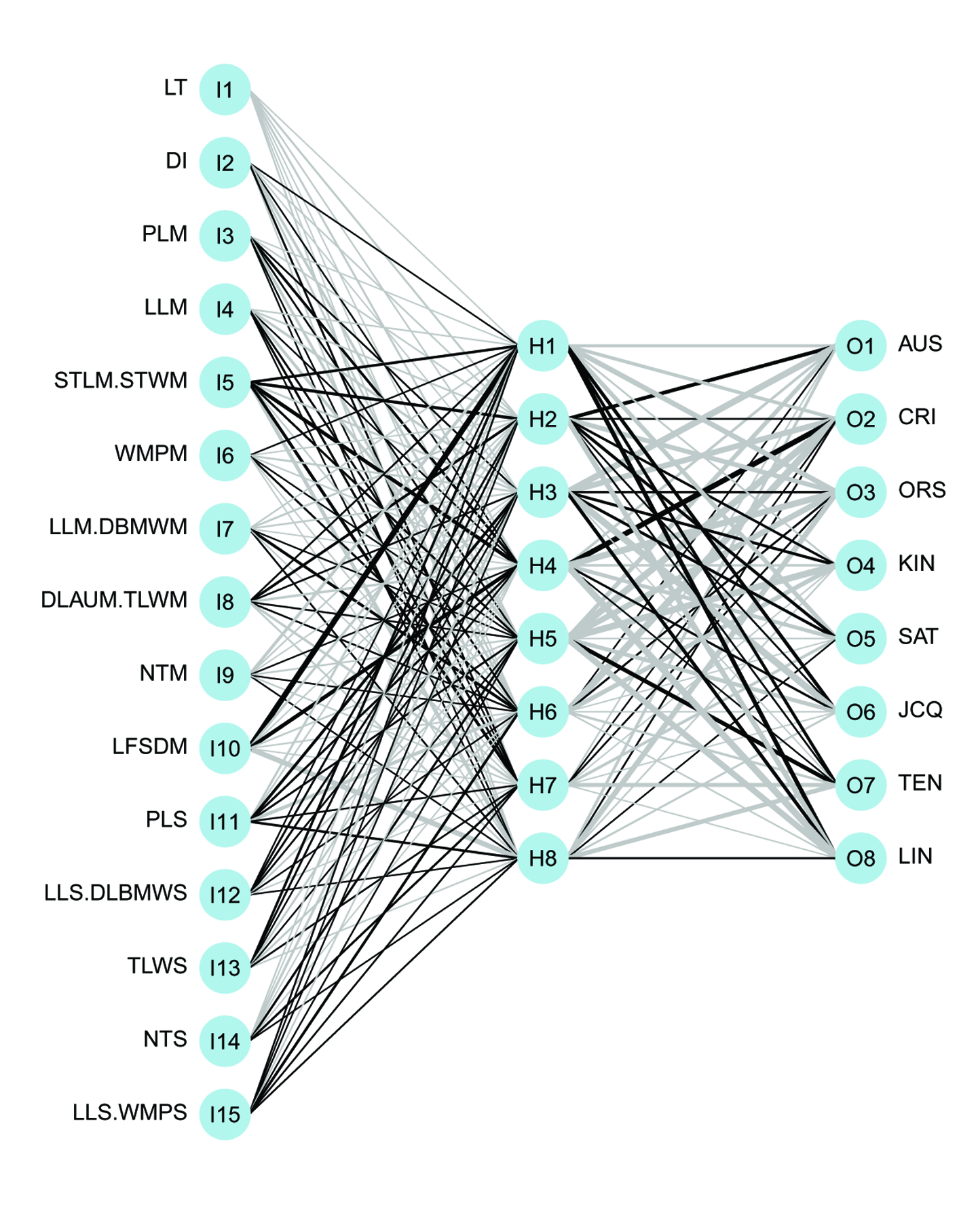

Supplement: S1 Fig — (I) input layers = 15; (H) hidden layers = 1; number of neurons = 8; (O) output layers = 8. Output layers correspond to taxa (see Table 1 for abbreviations), input layers correspond to variables (see Table 2 for abbreviations). Positive and negative connections are represented by black and grey lines, respectively. Line width indicates the strength of the connection. (TIF) [file pone.0199818.s004.tif]

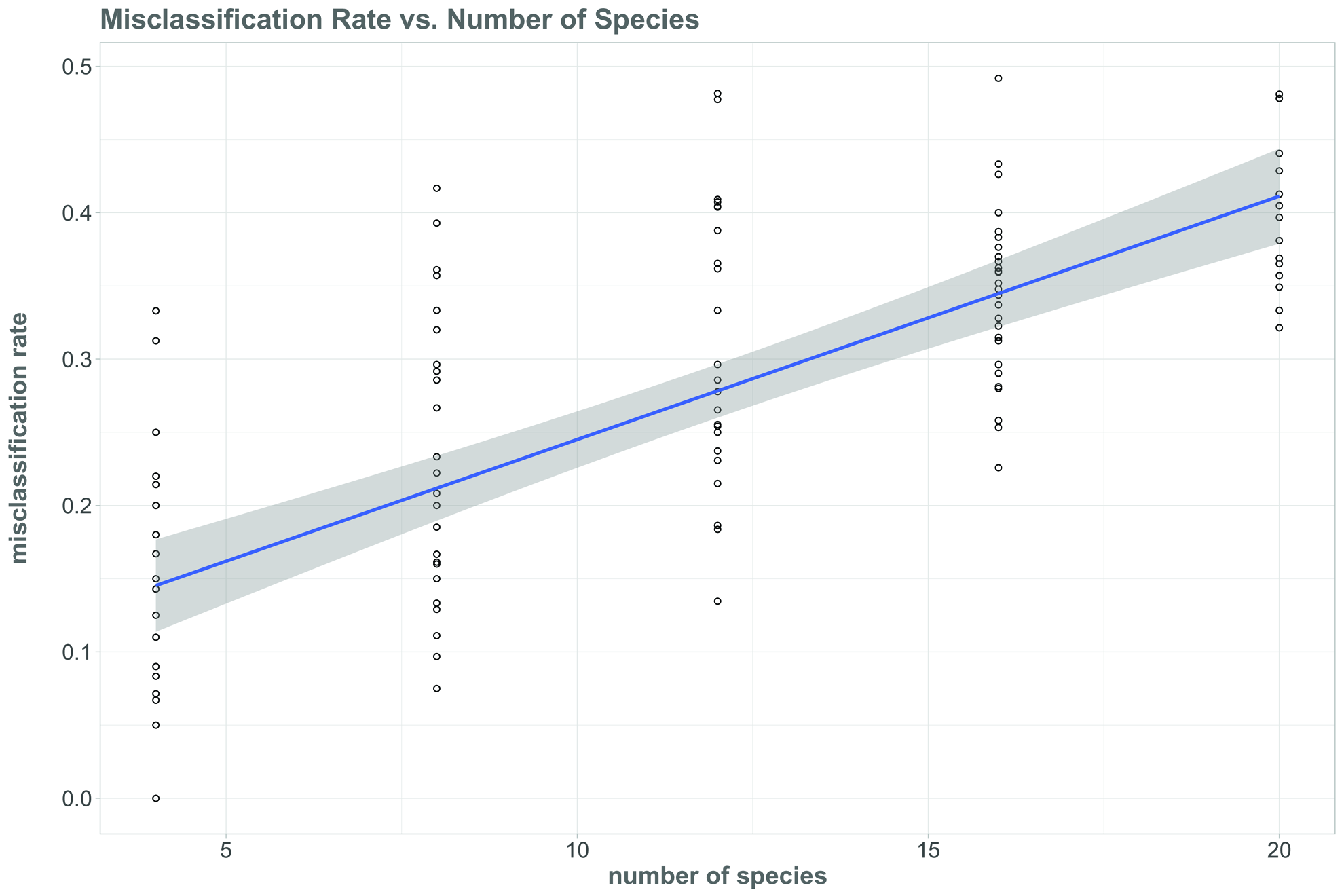

Supplement: S2 Fig — Misclassification rate in relation to the number of output layers (i.e., number of species and subspecies). Each point represents a different combination of randomly chosen OTUs. (TIF) [file pone.0199818.s005.tif]

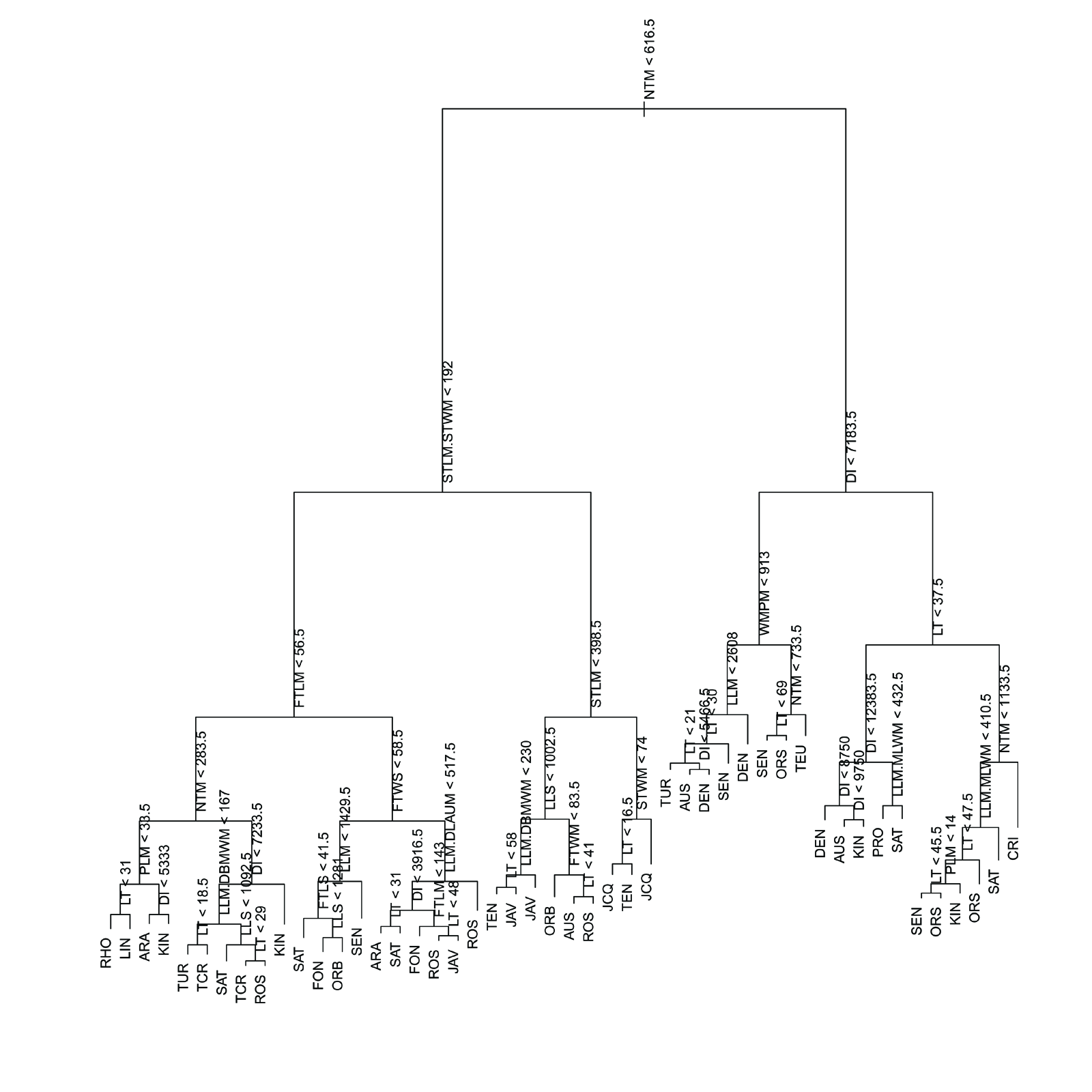

Supplement: S3 Fig — (TIF) [file pone.0199818.s006.tif]

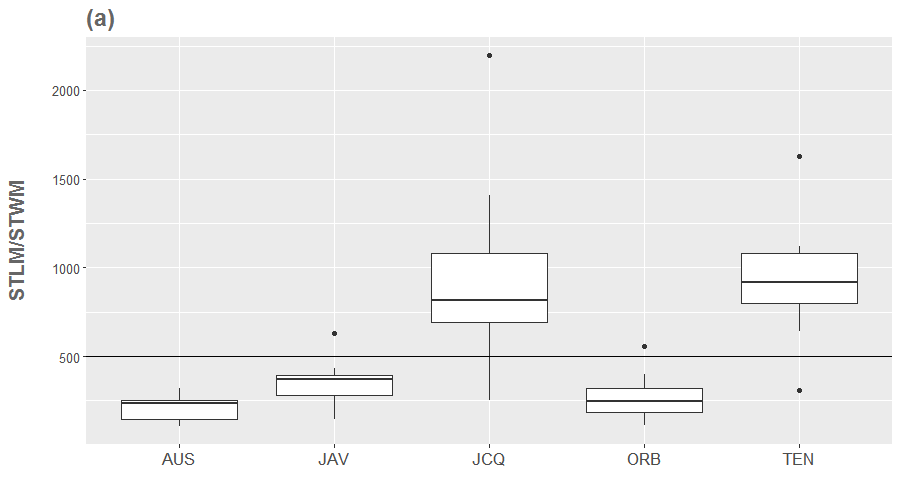


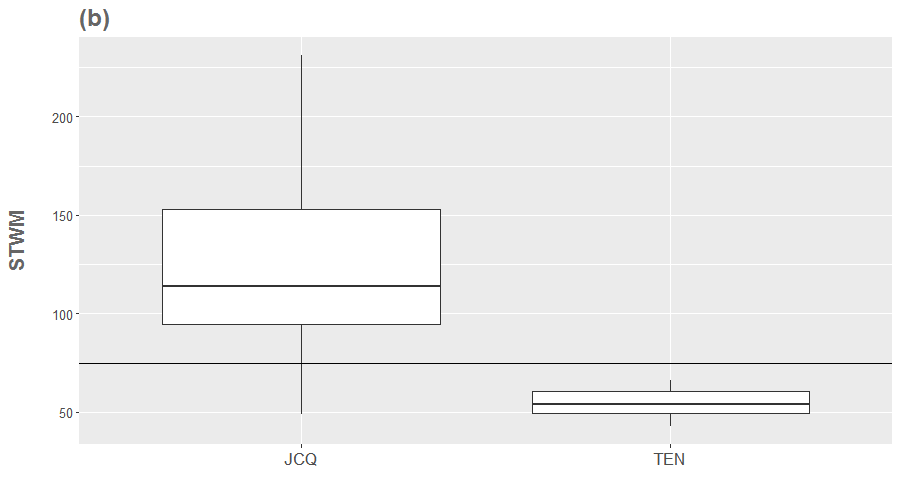


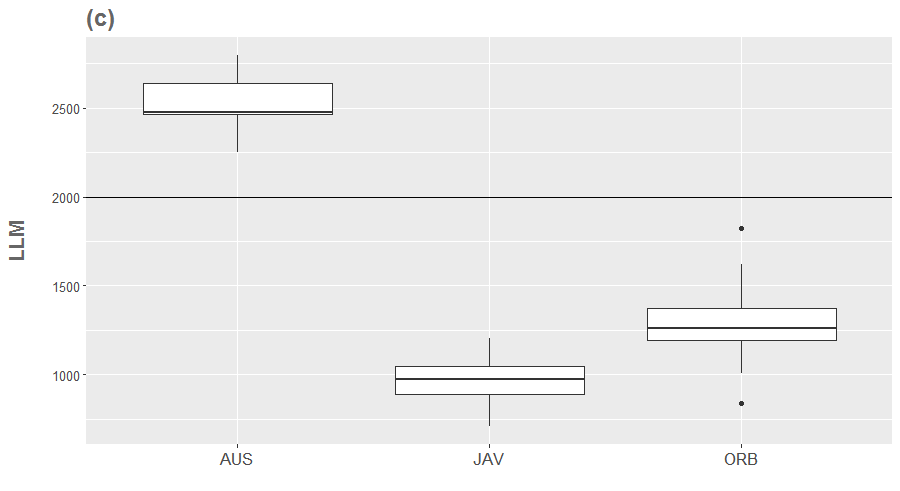


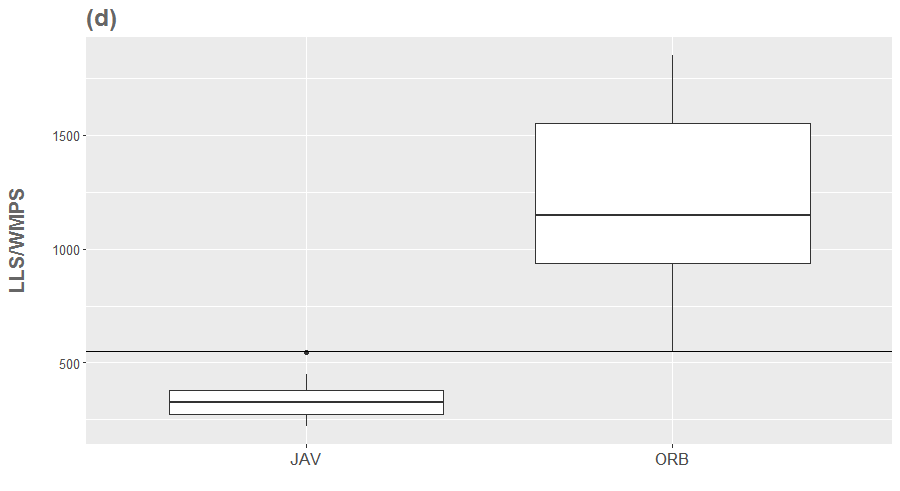


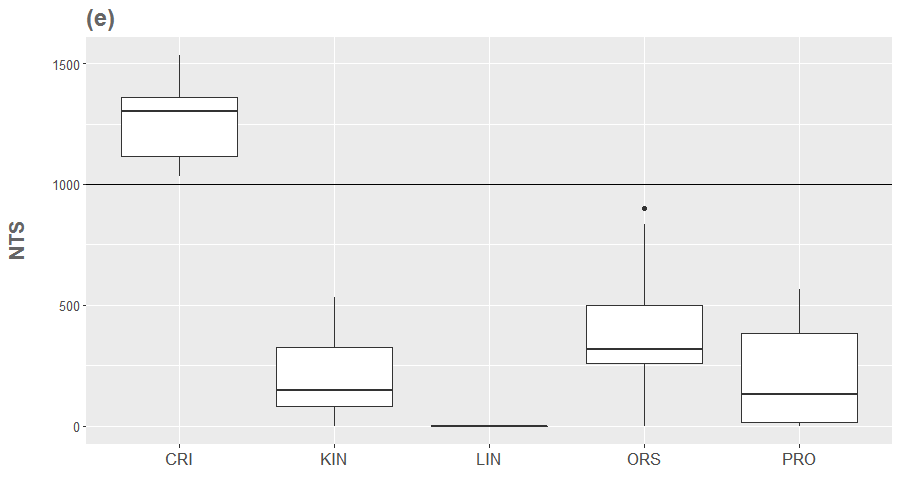


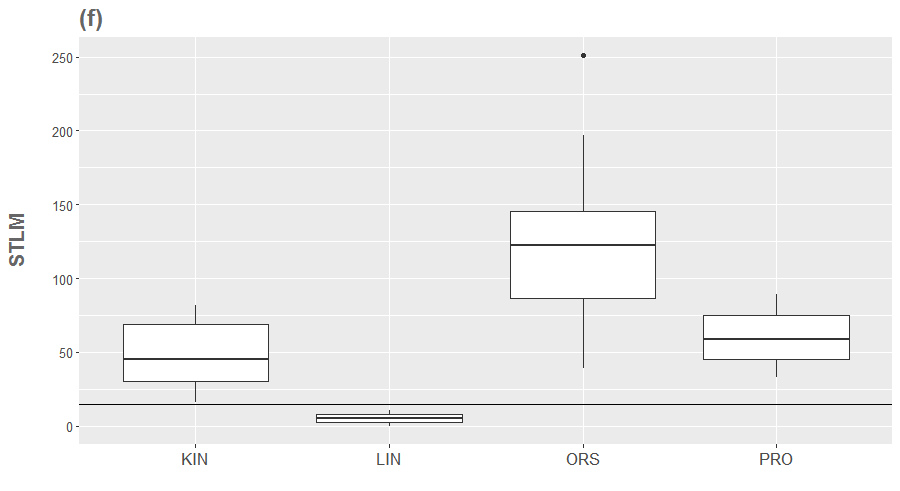


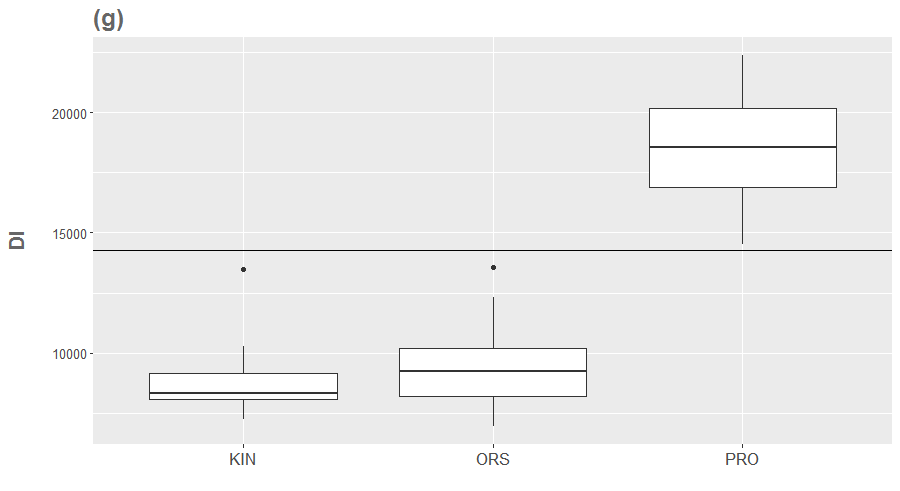


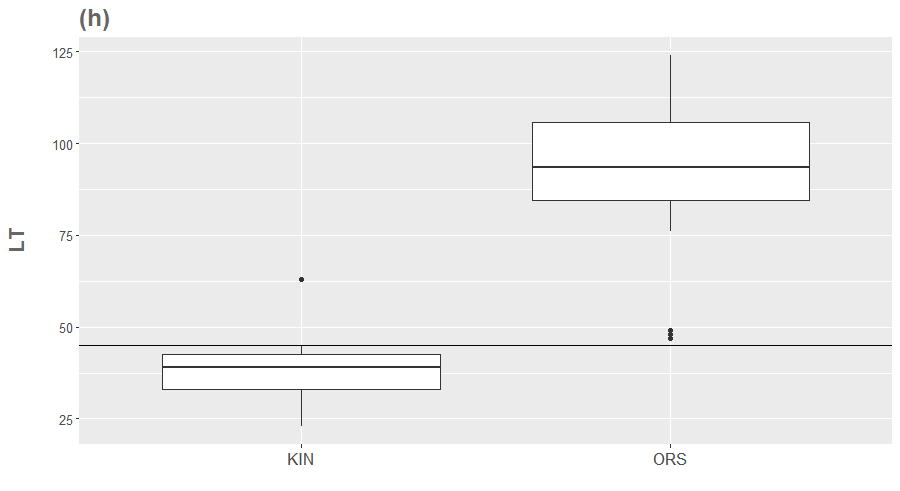


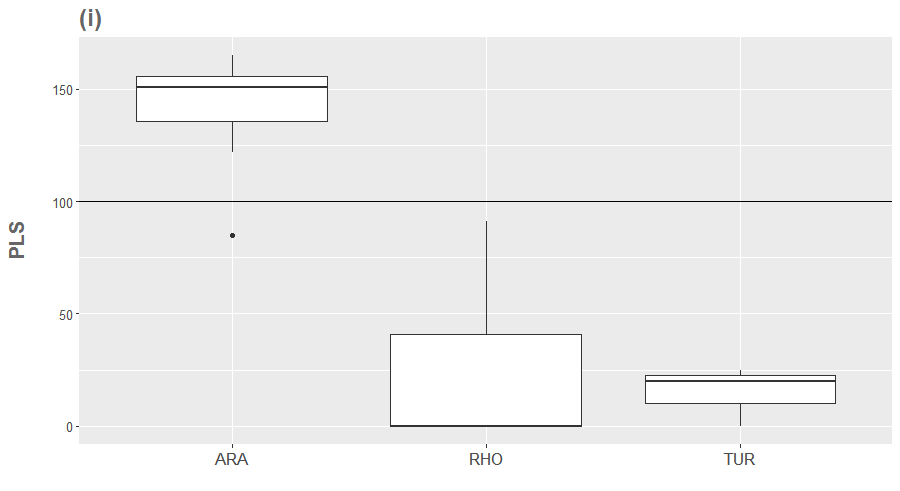


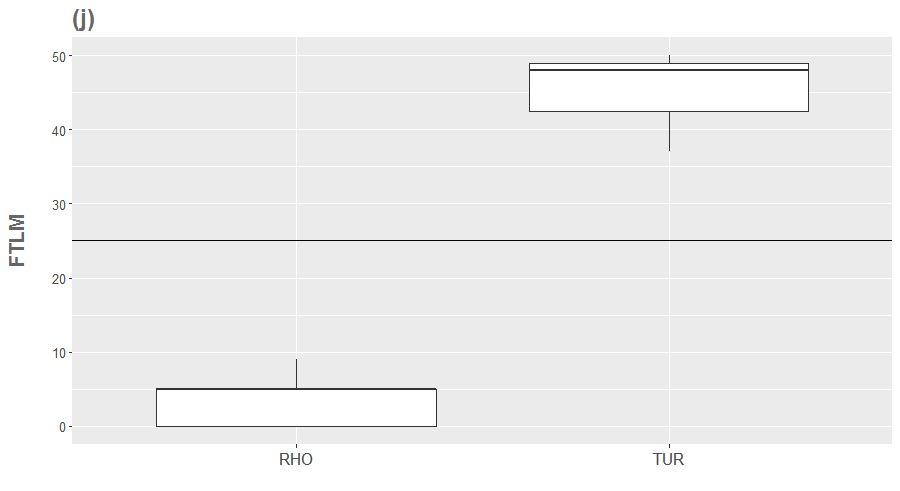


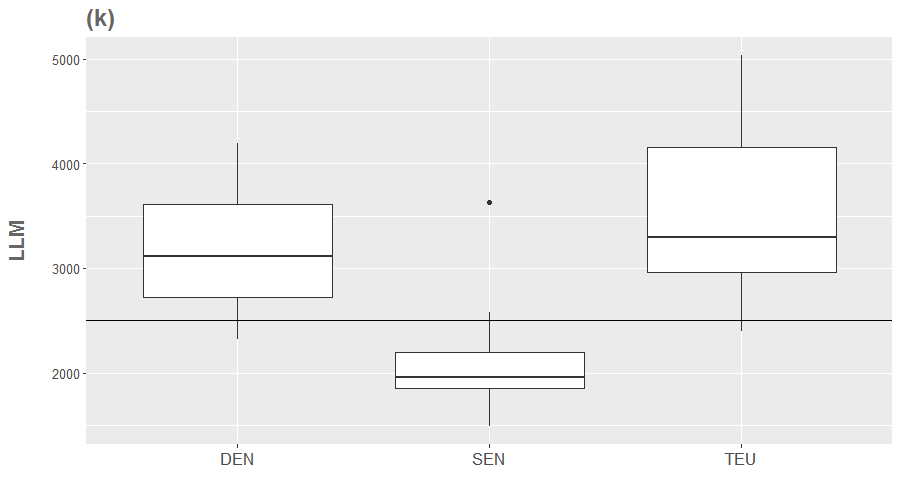


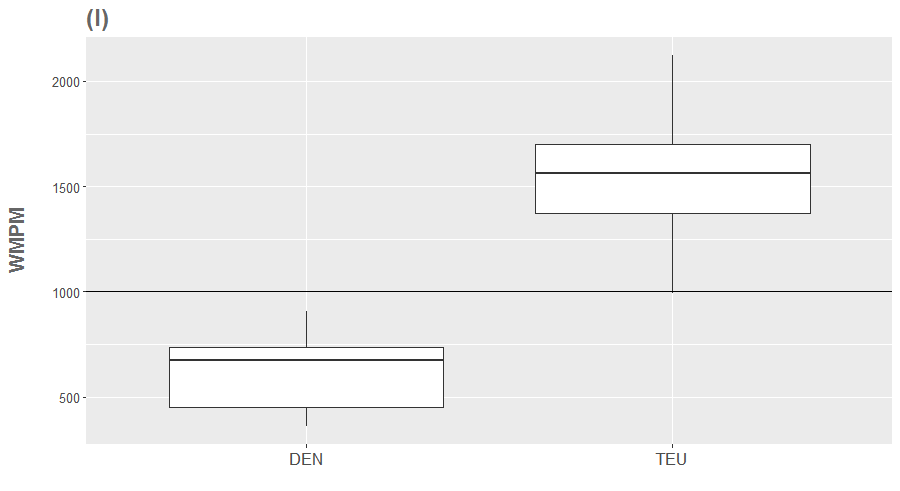


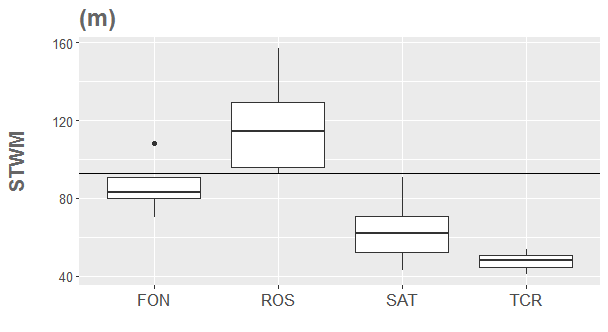


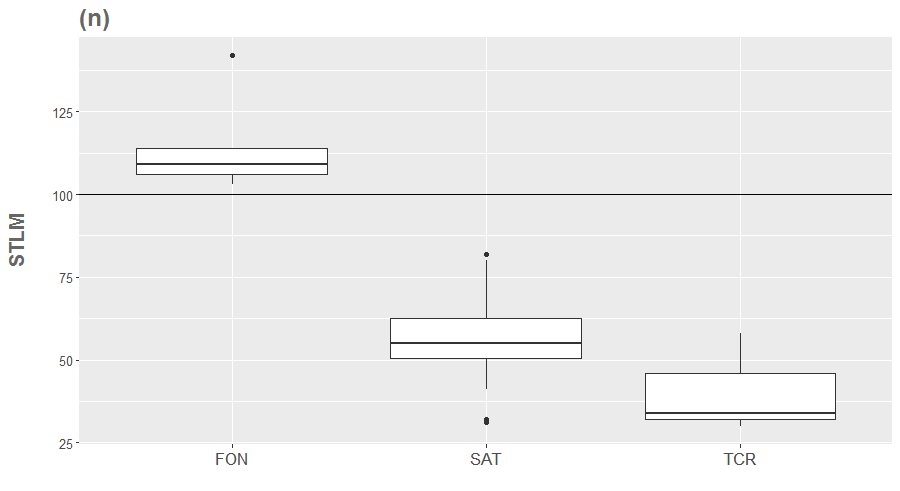


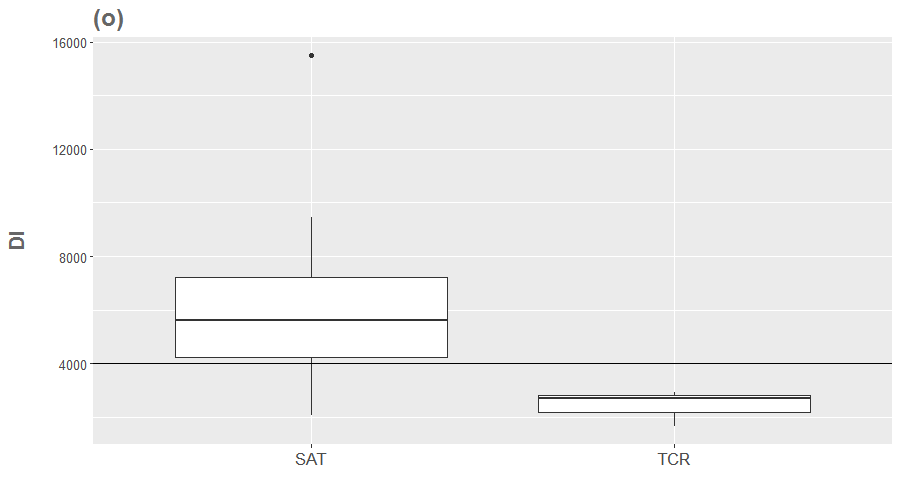

Supplement: S1 File — The black line in each box-plot indicates the threshold applied to perform the division. (DOCX) [file pone.0199818.s007.docx]

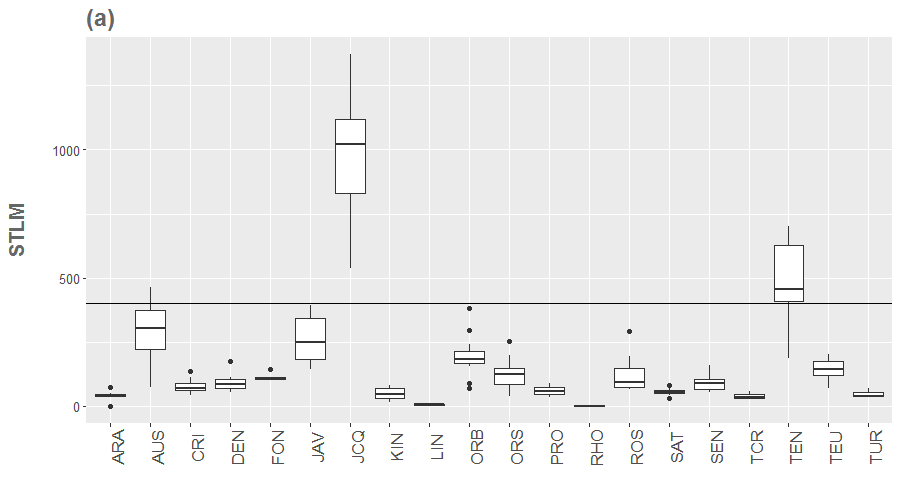


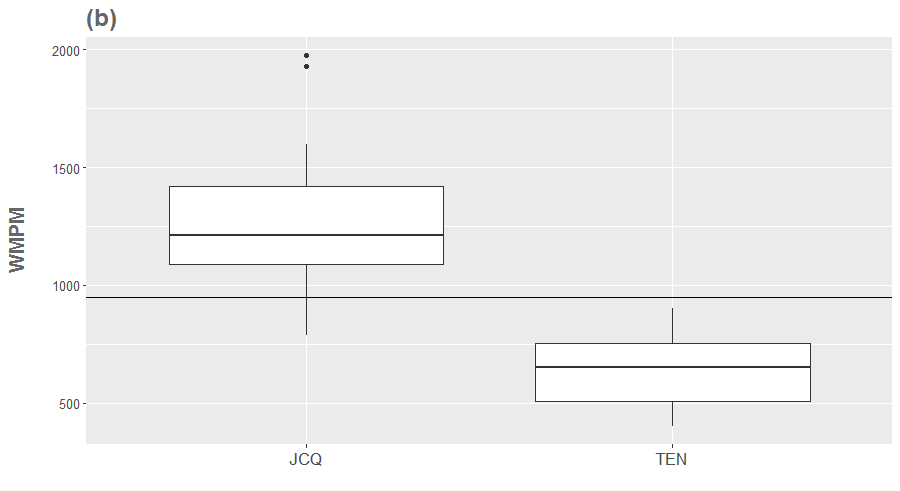


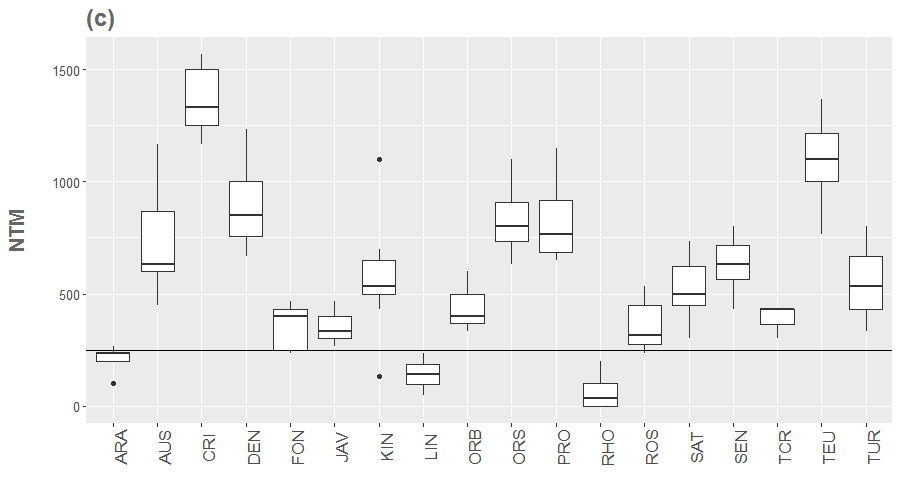


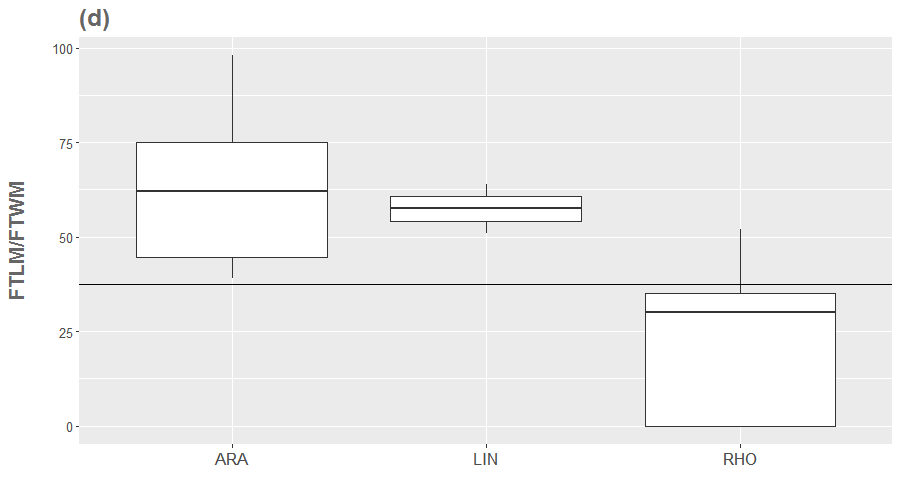


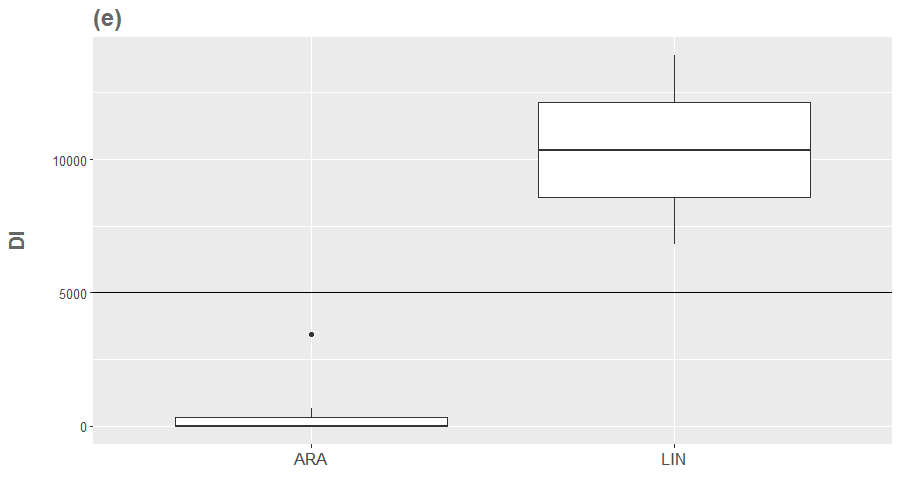


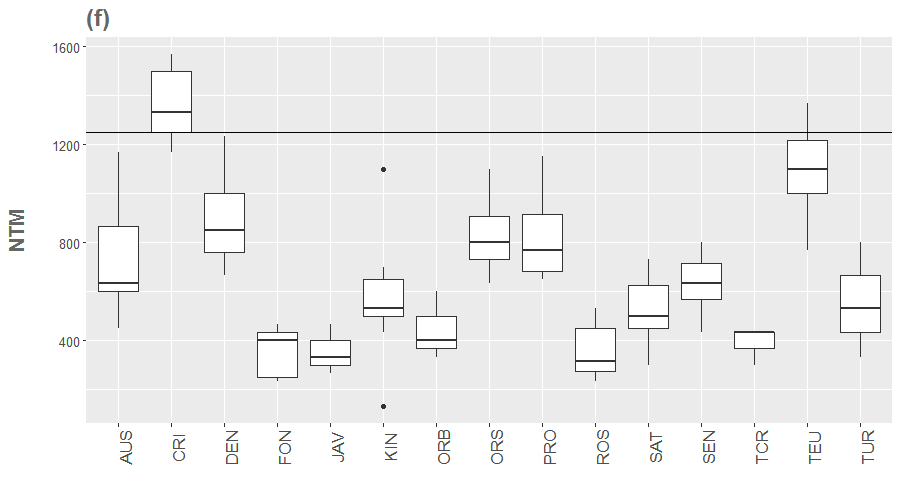


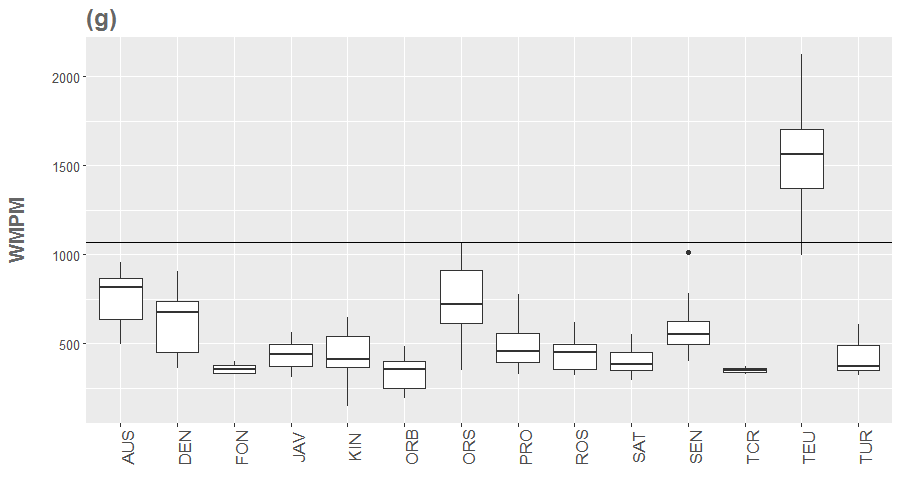


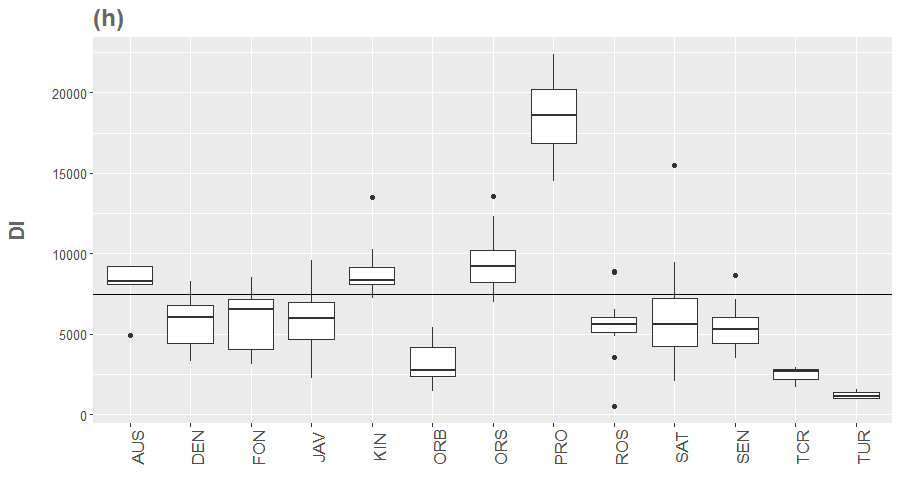


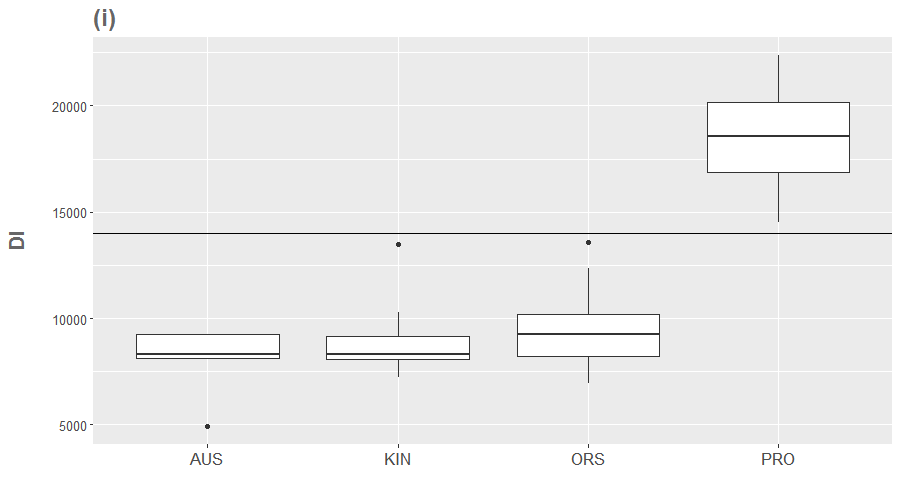


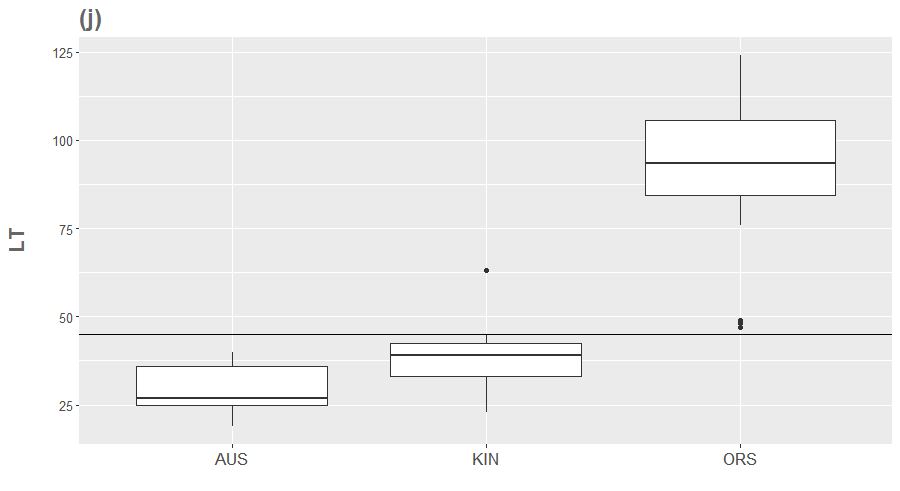


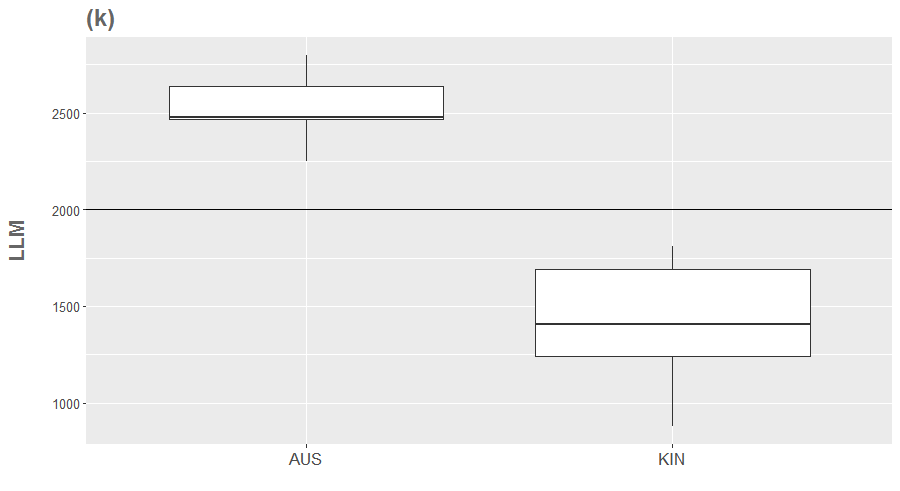


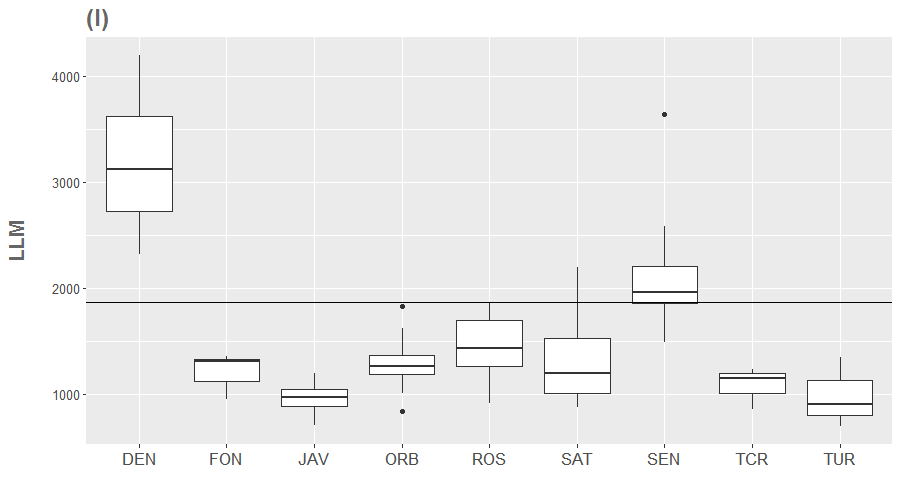


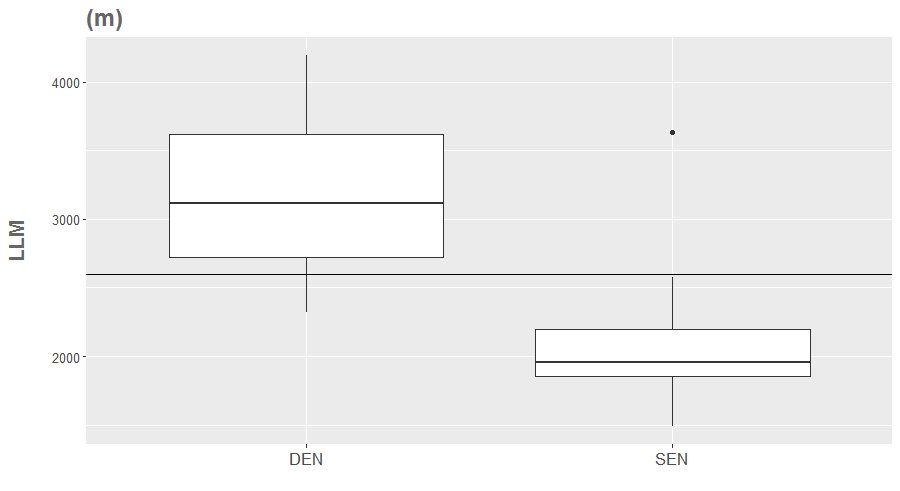


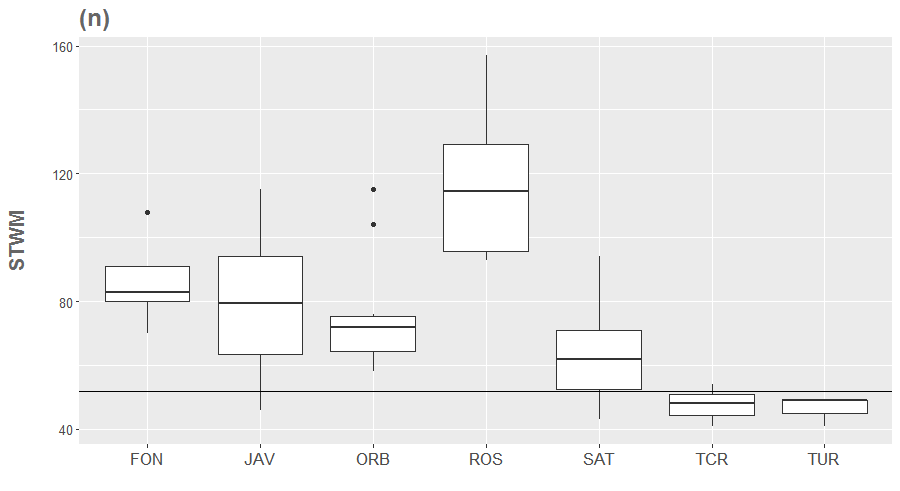


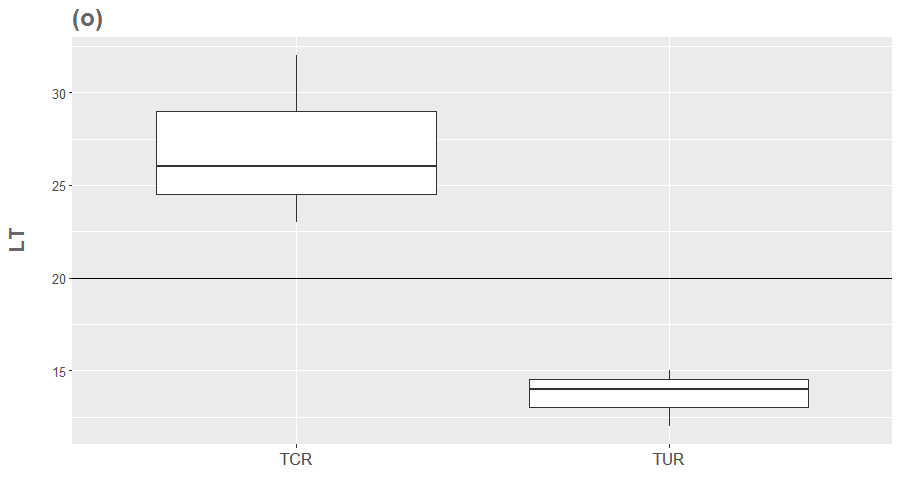


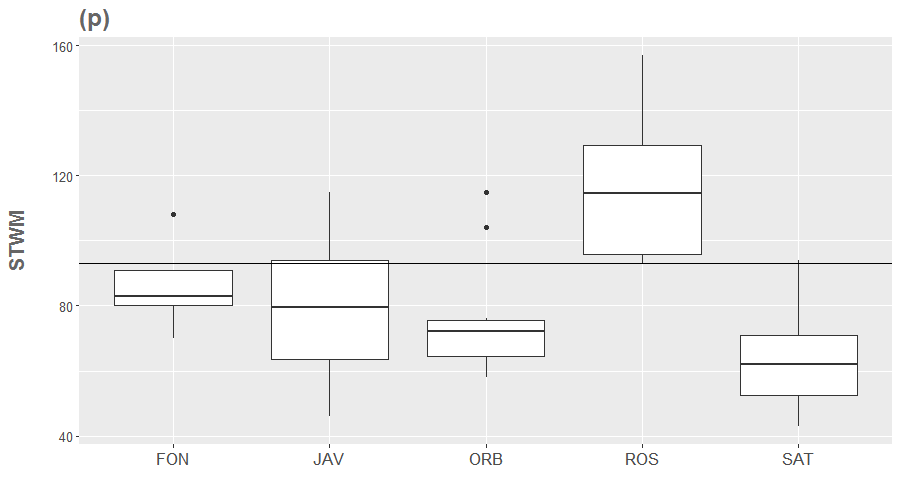


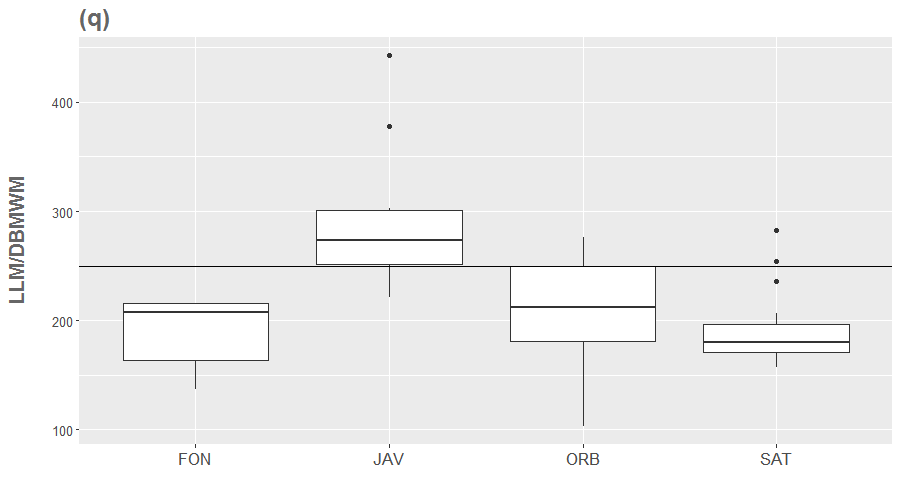


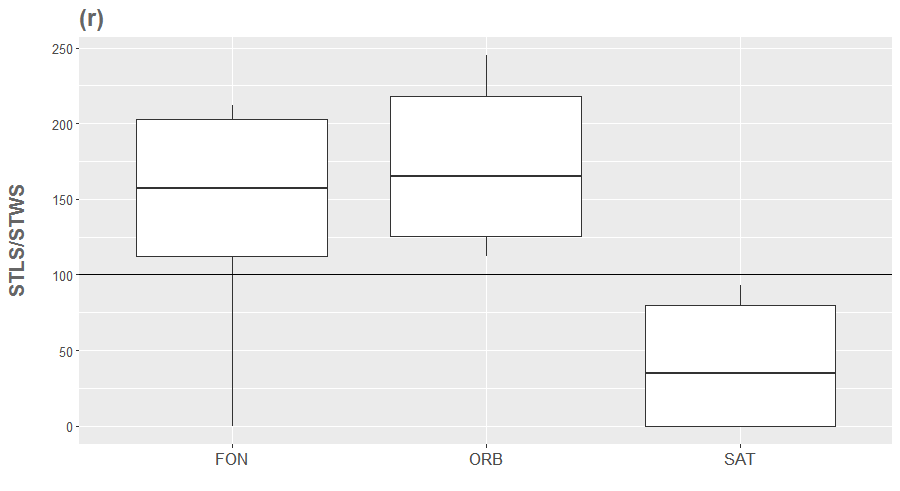


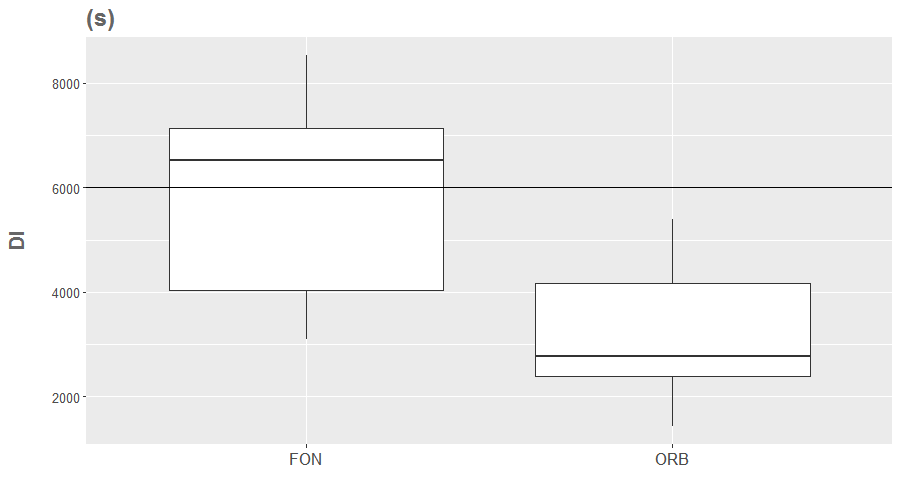

Supplement: S2 File — The black line in each box-plot indicates the threshold that minimizes the misclassification rate. (DOCX) [file pone.0199818.s008.docx]
